# New allosteric modulators of molecular chaperone TRAP1 from the integration of computational biology, medicinal chemistry, and biophysics

**DOI:** 10.1016/j.cstres.2026.100162

**Published:** 2026-02-26

**Authors:** Federica Guarra, Denis Komarov, Andrea Ciamarone, Luca Torielli, Viola Previtali, Natasha Margaroli, Elisa Romeo, Martina La Spina, Francesca Sbuelz, Claudio Laquatra, Marina Veronesi, Marco Lolicato, Cristina Arrigoni, Elisabetta Moroni, Stefano A. Serapian, Stefania Girotto, Andrea Rasola, Giorgio Colombo

**Affiliations:** 1 Department of Chemistry, University of Pavia, via Taramelli 12, 27100 Pavia, Italy; 2 Department of Biomedical Sciences, University of Padova, Padova, Italy; 3 Computational and Chemical Biology Department, Italian Institute of Technology, Via Morego 30, 16163, Genova, Italy; 4 Structural Biophysics Facility, Istituto Italiano di Tecnologia, via Morego 30, 16163, Genova, Italy; 5 Department of Molecular Medicine, University of Pavia, Via Ferrata 2, 27100 Pavia, Italy; 6 National Research Council of Italy, Istituto di Scienze e Tecnologie Chimiche “Giulio Natta” (SCITEC-CNR), Via Mario Bianco 9, 20131 Milano, Italy

**Keywords:** Allostery, Chaperones, TRAP1, Rational design, Molecular dynamics

## Abstract

Protein homeostasis is one of the key mechanisms that determine cellular life, and the Hsp90 family of molecular chaperones plays a key role in it. While Hsp90 dysregulation is a hallmark of numerous diseases, ranging from cancer to neurodegeneration, traditional inhibitors targeting its highly conserved ATPase site have largely failed in the clinic due to off-target toxicity and compensatory stress responses. One of the challenges in drug discovery, as well as in the development of chemical tools to investigate the specific roles of single family members, lies in achieving isoform specificity across the cytoplasm, endoplasmic reticulum, and mitochondria.Here, we exploit the intrinsic asymmetry of mitochondrial isoform TRAP1 and combine it with a fragment-design inspired approach to develop new possible TRAP1 targeting leads. We start from the consideration that the TRAP1 catalytic cycle relies on a strained, asymmetric dimer conformation that enforces sequential ATP hydrolysis. By integrating advanced computational dynamics with biochemical profiling, we demonstrate that small molecules can be rationally designed to target these transient asymmetric states. Our findings reveal that targeting allosteric, symmetry-breaking interfaces allows for the modulation of TRAP1, offering a novel platform and starting point for next-generation, isoform-specific anticancer therapeutics.

## Background

Correct protein homeostasis (proteostasis) is fundamental for the proper development and maintenance of all aspects of cell life. Hsp90 proteins are a family of ATPase-regulated molecular chaperones that oversee the key aspects of proteostasis by controlling the folding and activity of a plethora of “client” proteins (https://www.picard.ch/Hsp90Int/). Many of these clients have important roles in cell signaling, growth, metabolism, and intracellular communication. Dysregulation of these processes drives neoplastic transformation, and a marked overexpression of Hsp90 family proteins is frequently associated with neoplastic transformation in many tumor types.^1^, ^2^

Considering their central role in most neoplastic processes, Hsp90 chaperones have become an attractive anticancer pharmacological target.^1^, ^3^ As ATPase is key in regulating the functional dynamics of Hsp90 chaperones, most Hsp90 inhibitors are designed to compete with the naturally abundant ATP for the active site of the chaperone. Importantly, the therapeutic relevance of Hsp90 in cancer does not stem from recurrent mutations in the chaperone itself, but rather from variation in its functional engagement in stabilizing oncogenic and metastable client proteins, which renders tumor cells particularly dependent on Hsp90 activity. However, as this is highly conserved among all family members, these compounds impact indiscriminately on the functions of all Hsp90 proteins, also in healthy cells,^4^ and often induce a pro-survival heat shock response with the overexpression of Hsp70. It is therefore no surprise that dosage, toxicity, and tolerance issues have hampered their clinical progress and that, despite 17 active-site directed candidates made it to clinical trials^5^ (www.clinicaltrials.gov), none has been approved for use in human therapy, with only one that has recently emerged as an approved cancer drug in Japan.^6^, ^7^

In this context, the development of isoform-specific chemical tools, which could be further developed into therapeutically actionable inhibitors or used as probes to disentangle the complexities of chaperone networks, would be of paramount relevance.^8^ Indeed, different Hsp90 isoforms exist in different cell compartments: Hsp90 in the cytoplasm, Grp94 in the endoplasmic reticulum (ER), and TRAP1 in mitochondria.^9^ Importantly, their chaperoning roles do not overlap:^9^ Hsp90 clients are related to signal transduction, cell maintenance, growth, and invasiveness; Grp94 clients include IgGs and Toll-like receptors;^10^ TRAP1 clients are proteins related to mitochondrial homeostasis.^11^

Structurally, Hsp90s are homodimers, with each chain consisting of three globular domains, the N-terminal (NTD), Middle, and C-terminal (CTD). ATP binding at the NTD shifts the chaperone into a closed conformation that is significantly strained at the Middle:CTD interface, where clients bind. ATP hydrolysis is coupled with client remodeling: it relieves strain through rearrangement of the client binding-site residues, driving structural changes in the client. Biochemical and Cryo-EM data show that co-chaperones recruitment regulates the process by structurally organizing complexes for client activation (e.g., Cdc37, HOP), and by enhancing (e.g., Aha1) or slowing (e.g., p23) ATPase rates.^2^

Interestingly, although structural symmetry is often considered a hallmark of homo-oligomeric proteins, growing evidence indicates that controlled asymmetry can be an essential and exploitable feature of their function, enabling mechanistic behaviors that symmetric assemblies cannot achieve. In TRAP1, a catalytically active closed dimer adopts a highly strained asymmetric conformation, generated by a helix swap at the middle-C-terminal domain interface—a region that is broadly conserved and central to client recognition.^12^, ^13^ This intrinsic asymmetry is not merely structural: it redefines the ATPase cycle, producing differential hydrolysis propensities between protomers and enforcing sequential, deterministic ATP hydrolysis rather than cooperative or independent turnover.^14^, ^15^ Strikingly, hydrolysis of the first ATP is accompanied by a conformational flip of the asymmetric state, while the dimer remains closed for a second hydrolysis event, suggesting a direct coupling between energy expenditure and dynamic reorganization of client-binding sites. These findings highlight asymmetry as a programmable molecular feature that can drive ordered, energy-dependent remodeling steps.^14^, ^16^, ^17^

Here, building on the observation that asymmetry offers a powerful conceptual framework for the development of molecular probes, we ask whether it is possible to rationally design drug-like small molecules that can interact with and modulate transient asymmetric conformations.

To make progress along this route, we start from our recently discovered allosteric TRAP1 selective inhibitors, which indeed proved able to blunt in vitro tumorigenicity of highly malignant cells.^18^, ^19^ We combine advanced computational methods for the inclusion of functional protein dynamics information in the design of ligands with a range of biochemical and biophysical assays^20^, ^21^ to show that designed small molecules are indeed able to impact TRAP1 structural dynamics and modulate asymmetric ATP processing.

## Materials and methods

### Modeling of compound 1 binding to TRAP1

**Fig. 1:**
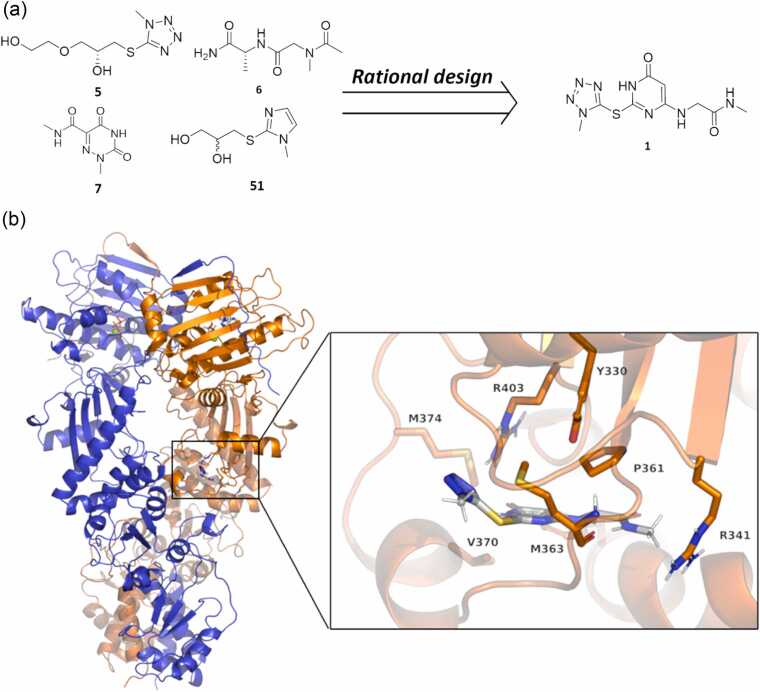
(a) Chemical structures of compounds **5**, **6**, **7** and **51** from refs.^18^, ^22^ and of compound **1** from this work. (b) Depiction of TRAP1(*cryst*)+**1** pose (see the main text for nomenclature) obtained from induced-fit docking calculations and selected as the starting point for MD simulations. A zoomed view of **1** in the allosteric pocket is also shown. Numbering of protein residues is in line with PDB code 4IPE. Details on pocket residues are in Materials and Methods.

**Preparation of TRAP1 crystal structure (TRAP1(*cryst*)).** TRAP1 crystal structure was retrieved from the Protein Data Bank (PDB code 4IPE^13^). Missing loop regions were modeled as reported previously,^18^, ^22^ resulting in the reconstruction of each TRAP1 protomer from residue Thr85 to His719. After removing all Co^2+^ cations, the AMP-PNP ligand was edited to ATP by replacing the N_β_-H group with oxygen. Hydrogens were then added employing the utility Reduce (AmberTools21),^30^ while protonation/tautomerization states were assigned using propka3,^31^ with Histidine residues modeled as described in published reports.^22^ The most abundant conformer was selected in the case of side chains with alternate locations; accordingly, Cys516 and Cys542 were modeled in the reduced oxidation state with no disulfide bridge. Additionally, the N- and C-termini were capped with acetyl (ACE) and N-methyl (NME) groups, respectively. Flexible docking calculations to allow adaptation of the pocket residues to the binding of compound **5** in Figure 1, were carried out as previously reported.^18^, ^22^

**Induced fit docking calculations.** Several conformations of TRAP1 in its closed, ATP binding state were initially employed for the calculations. One is the TRAP1(*cryst*) conformation directly obtained from the TRAP1 crystal structure (PDB code 4IPE) as detailed in the previous paragraph. Some other representative protein conformations were extracted from clustering of previous molecular dynamic simulations of TRAP1 in the presence of the allosteric inhibitor **5** from ref.^18^, ^22^ according to the clustering methods 1 and 2 described in ref.^22^

For the docking calculations, all protein structures were imported in Maestro (2021-4 release, Schrödinger Inc.) and converted to Maestro format employing the *Preprocess* utility in the *Protein Preparation Wizard* workflow. The *LigPrep* module in Maestro (vide supra) was employed to obtain energy-minimized 3D models of **1** with the OPLS4 force field.^32^ The tautomeric and ionization state of shown in Figure 1 was selected for the calculations after ranking of possible tautomeric and ionization states at pH 7.0 ± 2.0 based on the predicted abundance and overall state penalty. The *Induced Fit Docking* workflow was employed for calculations, keeping default settings including refinement of protein residues within 5 Å of the ligand poses and side chain optimization but setting Glide redocking mode to XP precision. The box defining the allosteric binding site was centered on the centroid of the following residues in protomer B: Arg341, Tyr342, Val360, Ser366, Asp369, Val370, Met374, Arg400, Phe401, Arg454, Phe456, Asp458, Tyr459, and Phe462 (numbering according to the 4IPE crystal structure). Docking poses were ranked for both starting protein conformations using multiple scoring functions (docking score, Glide GScore, Glide Emodel, Prime Energy, and IFD score). Among the top-scoring poses, the most representative ones—based on ligand conformation and orientation within the pocket—were selected as starting structures for subsequent all-atom molecular dynamics simulations. The structures of TRAP1 and TRAP1+**1** complexes are available as Supplementary Files at https://doi.org/10.5281/zenodo.18630105. TRAP1+**1** structures are identified as TRAP1(*cryst*)+**1** and TRAP1(*pocket*)+**1** which underwent MD simulations resulting in two distinct meta-trajectories. TRAP1(*cryst*) refers to the TRAP1 protein structure directly obtained from PDB code 4IPE as described above. TRAP1(*pocket*) corresponds to the second most populated cluster obtained using clustering method 2 in.^22^ Briefly, an average structure was calculated over the entire meta-trajectory of the TRAP1 in the presence of inhibitor **5** in Figure 1; residues within 6 Å from the ligand were included in the clustering, considering only heavy atoms of the side chain.

### Molecular dynamics (MD) simulations

**Force field parametrization and system preparation.** Protein residues were parameterized using the Amber forcefield ff14SB.^33^ For ATP we applied the parameters established by Meagher and coworkers,^34^ while those for Mg^2+^ were reported by Allnér et al.^35^ Tleap (AmberTools21)^30^ was used to build a truncated octahedral box of the TIP3P water model,^36^ ensuring a minimum distance of 12.5 Å between any protein atom and the nearest edge. Na^+^ ions, modeled employing parameters by Joung and Cheatham,^37^ were added to neutralize the box charge.

**Compound 1 parametrization.** The procedure involved use of the *antechamber* and *parmchk2* utilities from AmberTools21.^30^ GAFF2 parameters were assigned to describe bonded terms and non-bonded Lennard-Jones interactions. Atomic partial charges were derived exploiting *Gaussian16*^38^ in conjunction with *antechamber*. First, compound **1** was geometry-optimized, and vibrational frequency calculations were performed at the B3LYP/6–31 G(d,p) level of theory. The electrostatic potential was then computed at the Hartree-Fock/6–31 G(d,p) level of theory and sampled on 10 concentric shells per atom with a density of 17 grid points per square Bohr. Finally, atomic point charges were obtained by RESP fitting using *antechamber.*^39^

**MD simulations protocol.** Molecular dynamics simulations were run employing the *sande*r and *pmemd* engines from AmberTools (version 21) and Amber (version 20),^40^ respectively. *Sander* was employed during the initial stages of minimization, solvent equilibration, and heating, while the GPU-accelerated *pmemd.cuda* engine was used for the subsequent stages of equilibration and production. Four independent replicas were run for each system, which included two minimization phases, heating, system equilibration, and production.

In the first minimization, 500 steps of steepest descent followed by 500 steps of conjugate gradient minimization were performed. Heavy atoms of aminoacids, ATP, and allosteric ligands, together with Mg^2+^ ions were constrained using a harmonic positional restraint with a force constant of 500 kcal mol^−1^ Å^−2^. In the second minimization step, the full system was allowed to relax through 1000 steepest descent steps and 1500 conjugate gradient steps.

Minimization was followed by a rapid heating from 0 to 300 K (20 ps; NVT ensemble) in which positions of non-hydrogen atoms of protein residues, ATP and ligands, and Mg^2+^ were gently restrained with a 5 kcal mol^−1^ Å^−2^ constant. Temperature control was enforced via the Langevin thermostat^41^ with a weak coupling (0.75 ps^−1^ collision frequency). SHAKE constraints for bonds containing hydrogens were introduced and kept thereafter.^42^ Accordingly, the time step was set to 2 fs and will remain so for the rest of the simulation. The cutoff for non-bonded interactions for both minimization and heating was 10 Å shifting thereafter to the particle mesh Ewald method^43^ (Coulomb interactions computed in direct space up to 12 Å).

Following heating, the system was equilibrated at 300 K with no restraints for 4 ns in the NpT ensemble to allow for density adjustment. Pressure control was enforced through the Berendsen’s barostat with a 2 ps relaxation time, while coupling to the Langevin thermostat was tightened (2 ps^−1^ collision frequency). During this phase, the non-bonded interaction cutoff was maintained at 10 Å, while Coulomb interactions were computed in direct space up to 15 Å.

Finally, MD production was carried out in the NpT ensemble. Each system was simulated for 1 μs per replica employing the same parameters of NpT equilibration at 300 K, except that the non-bonded interaction cutoff was shortened to 8 Å (Coulomb interactions computed in direct space up to 10 Å). Coordinates were stored every 50 ps, resulting in 20,000 frames per replica.

For each TRAP1 system, MD trajectories from the four replicas were concatenated to form meta-trajectories using *cpptraj* (AmberTools 21); in doing so, we centered and imaged the trajectory, stripped solvent and Na^+^ ions, and aligned all structures to the first frame in the first replica using, as a reference, the backbone atoms of protomer B residues involved in secondary structures. As a result, meta-trajectories of 80,000 frames covering a time interval of 4 μs were obtained for each system.

**Clustering of MD meta-trajectories.** Prior to the analysis TRAP1(*cryst*)+**1** and TRAP1(*pocket*)+**1** meta-trajectories were aligned on pocket residues, defined as those within 6 Å from ligand atoms in the average structure calculated from heavy atom positions along each meta-trajectory. Clustering analyses were carried out with *cpptraj* (AmberTools21) employing a hierarchical agglomerative algorithm with complete linkage on one every two frames, stopping at 6 clusters and considering only ligand and pocket residues’ side chains heavy atoms. Pocket residues are defined as those within 6 Å of compound **1** in the average structures extracted from each meta-trajectory. Cluster structures are available as Supplementary Files at https://doi.org/10.5281/zenodo.18630105. Clusters’ populations are listed in Table S1.

### Docking screening of pyrimidone and triazine derivatives

**Receptor and ligand structure preparation.** Two different protein conformations were considered for binding pose predictions. One is TRAP1(*cryst*), mentioned earlier in the main text; the second protein conformation (TRAP1(*MD*)) was obtained from previously reported MD simulations of TRAP1 with compound **5** in ref.^22^ and is the centroid of the most populated cluster resulting after considering the backbone of all residues in protomer B (containing the pocket) and loop regions of protomer A as described in the work by Triveri et al.^22^ (clustering method 1). The structure was prepared following a protocol analogous to the one already described earlier in this section, imported in Maestro (2021-4 release, Schrödinger Inc.) and converted to Maestro format employing the preprocess utility in the *Protein Preparation Wizard* workflow.

As for compound **1**, ligand structures in Figure 3 were manually built in Maestro and prepared using the *LigPrep* module employing the OPLS4 force field. For each ligand possible tautomeric and ionization states at pH 7.0 ± 2.0 were generated and ranked based on the predicted tautomer abundance, ionization, and overall state penalty.

**Grid generation, docking calculations, and scoring.** Rigid receptor flexible ligand docking calculations of the newly built libraries were carried out employing Glide docking software^44^, ^45^ implemented in 2021-4 version of Maestro. The grid, needed for pose prediction and scoring, was generated keeping default settings with no Van der Waals radius scaling, and the enclosing box for the docking site was defined starting from the centroid of selected residues in the TRAP1 allosteric pocket already mentioned in the induced fit docking paragraph. The *Ligand Docking* workflow was employed for the actual calculations: first SP (Standard precision) mode calculations were run, followed by XP (Extra precision) docking of the obtained poses. In both cases 10,000 poses per ligand were kept for the initial phase of docking, and the best 1000 poses were retained for the minimization phase. A Van der Waals radius scaling factor of 0.80 was applied to atoms with partial charge lower than 0.15. Default settings were kept for all the other options. After docking, ligand poses were scored with Glide proprietary scoring functions, including Docking score and XP Gscore. Of these, the Docking score also considers penalties related to the ionization/tautomeric states in addition to the interaction energy, while the XP Gscore is specific to XP mode with additional terms and different treatments than the SP Gscore. A consensus approach was employed to select the most promising compounds. Accordingly, compounds that consistently displayed the best rankings based on both scoring criteria and in both protein conformations were prioritized for synthesis.

### Computational characterization of compound 4 binding to TRAP1

#### Molecular dynamics (MD) simulations

**Force field parametrization, system preparation, and MD protocol.** After visual analysis of poses predicted by the above-described docking calculations in SP and XP modes, the best pose of compound **4** predicted by SP docking in TRAP1(*cryst*) conformation was selected as the starting structure for MD simulations and is available as a Supplementary File at https://doi.org/10.5281/zenodo.18630105. For comparison, simulations of TRAP1(*cryst*) in the absence of allosteric ligand were also run.

Force field parametrization and system preparation were analogous to those employed for MD simulations of TRAP1+**1** complexes. Compound **4** was modeled in its negatively charged carboxylate ionization state. Four 500 ns long independent replicas were run with a protocol identical to the one already described in an earlier paragraph for each system.

MD trajectories from the four 500 ns long replicas were concatenated to form single meta-trajectories using *cpptraj* (AmberTools 21). In doing so, we centered and imaged the trajectory, stripped solvent and Na^+^ ions, and aligned all structures to the first frame in the first replica using, as a reference, the backbone atoms of residues involved in secondary structures. As a result, meta-trajectories of 40,000 frames covering a time interval of 2 μs were obtained for TRAP1(*cryst*) and TRAP1(*cryst*)+**4.**

**Distribution of inter-protomer distances and contacts.** All analyses were carried out with *cpptraj* on each system meta-trajectories. Domain subdivision was the following: NTD comprised residues 101-308; MD 311-571; CTD 587-719. For computing interprotomer distances along the meta-trajectories the center of mass of each protomer domain was considered. Inter-protomer contacts were computed with the *nativecontacts* command, employing a distance cutoff of 7 Å and considering only heavy atoms. Both native and non-native contacts were summed up, with no distinction. Distributions were plotted employing the *seaborn* and *matplotlib* Python packages.

### Synthesis of compounds 1-10

**General.** Solvents and reagents were obtained from commercial suppliers and used without further purification. NMR experiments were run on a Bruker Avance III 400 MHz spectrometer (400.13 MHz for 1H and 100.62 MHz for 13 C), equipped with a BBI probe and Z-gradients, or on a Bruker FT NMR Avance III 600 MHz spectrometer (600.130 MHz for 1H and 150.903 MHz for 13 C) equipped with a 5 mm CryoProbe QCI quadruple resonance, a shielded Z-gradient coil, and the automatic sample changer SampleJet NMR system. Spectra were acquired at 300 K, using deuterated dimethylsulfoxide (DMSO-*d*_6_) or deuterated chloroform (CDCl_3_-*d*) as a solvent. Chemical shifts for ^1^H and ^13^C spectra were recorded in parts per million using the residual non-deuterated solvent as the internal standard. The coupling constants of the splitting patterns were reported in Hz and were indicated according, that is, as singlet (s), doublet (d), triplet (t) etc or multiplet (m). UPLC−MS analyses were run on a Waters ACQUITY UPLC/MS system consisting of an SQD (single quadrupole detector) mass spectrometer equipped with an electrospray ionization interface and a photodiode array detector. The PDA range was 210-400 nm. The analyses were performed on either an ACQUITY UPLC HSS T3 C18 column (50 × 2.1 mm i.d., particle size 1.8 µm) with a VanGuard HSS T3 C18 precolumn (5 × 2.1 mm i.d., particle size 1.8 µm) (logD <1) or an ACQUITY UPLC BEH C18 column (50 × 2.1 mm i.d., particle size 1.7 µm) with a VanGuard BEH C18 precolumn (5 × 2.1 mm i.d., particle size 1.7 µm) (log D>1). The mobile phase was 10 mM NH_4_OAc in H2O at pH 5 adjusted with AcOH (A) and 10 mM NH_4_OAc in MeCN–H_2_O (95:5) at pH 5 (B). Electrospray ionization in positive and negative mode was applied in the mass scan range 100-500 Da. Methods and gradients used were the following: Generic method. Column: Waters ACQUITY UPLC BEH C18, 1.7 µm, 50 × 2.1 mm i.d. Linear gradient: 0−0.2 min, 5% B; 0.2−2.7 min, 5%-95% B; 2.7-2.8 min, 95%-100% B; 2.8-3.0 min, 100% B. Flow rate: 0.5 mL/min. Apolar method. Column: Waters ACQUITY UPLC BEH C18, 1.7 µm, 50 × 2.1 mm i.d. Precolumn: VanGuard BEH C18, 1.7 µm, 5 × 2.1 mm i.d. Gradient: 0−0.2 min, 50% B; 0.2-2.7 min, 50%-100% B; 2.7-3.0 min, 100% B. Flow rate: 0.5 mL/min. Compounds were named according to IUPAC convention. All final compounds displayed ≥95% purity as determined by UPLC/MS analysis.

### Synthesis of intermediates (*int_1a*, *int_1b*, *int_2*) and compound 1

**2,6-dibromopyrimidin-4-ol (*****int_1a).*** 2,4,6-tribromopyrimidine (200.0 mg, 0.6 mmol, 1.0 equiv.) was dissolved in dioxane (6.0 mL). NaOH (2 M in H_2_O) (0.3 mL, 0.6 mmol, 1.0 equiv.) was added, and the reaction was stirred for 4 h at room temperature. Solvent was then evaporated, and the crude residue purified by normal phase chromatography purification with a CombiFlash Rf Teledyne ISCO apparatus (12 g silica cartridge, solvent A: DCM, solvent B: MeOH, gradient 0 to 20% solvent B in 20 min) to yield the title compound as an off-white solid. (7.6 mg, 10%). ^1^H NMR (400 MHz, DMSO-*d*_6_) *δ* 5.61 ^13^C NMR (101 MHz, DMSO-*d*_6_) 175.63, 150.10, 144.56, 125.21 *t*_R_: 2.17 min (generic method). ESI-MS for C_4_H_2_Br_2_N_2_O: calculated 253.8, found *m/z* 252.8 [M-H]^-^ and 254.8 [M+H]^+^.

**4,6-dibromopyrimidin-2-ol (*****int_1b*****).** 2,4,6-tribromopyrimidine (200.0 mg, 0.6 mmol, 1.0 equiv.) was dissolved in dioxane (6.0 mL). NaOH (2 M in H_2_O) (0.3 mL, 0.6 mmol, 1.0 equiv.) was added, and the reaction was stirred for 4 h at room temperature. Solvent was then evaporated, and the crude residue purified by normal phase chromatography purification with a CombiFlash Rf Teledyne ISCO apparatus (12 g silica cartridge, solvent A: DCM, solvent B: MeOH, gradient 0 to 20% solvent B in 20 min) to yield the title compound as an off-white solid. (4.5 mg, 3%). ^1^H NMR (400 MHz, DMSO-*d*_6_) *δ* 6.01 ^13^C NMR (101 MHz, DMSO-*d*_6_) 158.24, 142.10, 112.20. *t*_R_: 2.12 min (generic method). ESI-MS for C_4_H_2_Br_2_N_2_O: calculated 253.8, found *m/z* 252.8 [M-H]^-^ and 254.8 [M+H]^+^.

**2-((2-bromo-6-hydroxypyrimidin-4-yl)amino)-N-methylacetamide (*****int_2*****).** Under nitrogen, *int_1a* (50.0 mg, 0.2 mmol, 1.0 equiv), 2-amino-N-methylacetamide hydrochloride (37.5 mg, 0.3 mmol, 1.5 equiv.), anhydrous TEA (0.1 mL, 0.8 mmol, 4.0 equiv.) were mixed in a Schlenk flask and then dissolved in dry ACN (0.5 mL). After heating the reaction heated at 90 °C for 12 h, the solvent was evaporated. The crude residue was purified by normal phase chromatography purification with a CombiFlash Rf Teledyne ISCO apparatus (12 g silica cartridge, solvent A: DCM, solvent B: MeOH, gradient 0 to 20% solvent B in 20 min) to yield the title compound as an off-white solid. (26.0 mg, 50%). ^1^H NMR (400 MHz, DMSO-*d*_6_) 12.01 (s, 1H), 7.70 (q, *J* = 4.8 Hz, 1H), 5.50 (s, 1H), 3.49 (s, 2H), 2.56 (d, *J* = 4.4 Hz, 3H). *t*_R_: 1.96 min (generic method). ESI-MS for C_7_H_9_BrN_4_O_2_: calculated 259.9, found *m/z* 258.9 [M-H]^-^ and 260.9 [M+H]^+^.

**2-((6-hydroxy-2-((1-methyl-1H-tetrazol-5-yl)thio)pyrimidin-4-yl)amino)-N-methylacetamide(1).** ***Int1b*** (13.0 mg, 0.05 mmol, 1.0 equiv.), 1-methyl-1H-tetrazole-5-thiol (11.6 mg, 0.2 mmol, 2.0 equiv.) and anhydrous TEA (25.0 μL, 0.4 mmol, 4.0 equiv.) were mixed in a Schlenk flask and dissolved in dry ACN (0.2 mL) under an inert nitrogen atmosphere. The reaction was heated at 120 °C for 32 h. Solvent was then evaporated, and the crude residue purified by normal phase chromatography purification with a CombiFlash Rf Teledyne ISCO apparatus (12 g silica cartridge, solvent A: DCM, solvent B: MeOH, gradient 0 to 20% solvent B in 20 min) to yield the title compound as an off-white solid. (2.96 mg, 20%). ^1^H NMR (400 MHz, DMSO-*d*_6_) 12.01 (s, 1H), 7.70 (q, *J* = 4.8 Hz, 1H), 5.50 (s, 1H), 3.99 (s, 3H), 3.49 (s, 2H), 2.56 (d, *J* = 4.4 Hz, 3H).^13^C NMR (101 MHz, DMSO-*d*_6_) 169.23, 163.93, 161.65, 156.01, 148.05, 98.74, 43.56, 34.89, 25.94. *t*_R_: 1.67 min (generic method). ESI-MS for C_9_H_12_N_8_O_2_S: calculated 296.1, found *m/z* 295.1 [M-H]^-^ and 296.1 [M+H]^+^.

### Synthesis of intermediate *int_3* and compounds 2–10

**2-((4-chloro-6-methoxy-1,3,5-triazin-2-yl)amino)-N-methylacetamide (*****int_3).*** Under nitrogen 2,4-dichloro-6-methoxy-1,3,5-triazine (180.0 mg, 1.0 mmol, 1.0 equiv.) was dissolved in THF (4.0 mL) and cooled to −78 °C. A solution of 2-amino-N-methylacetamide hydrochloride (162.5 mg, 1.3 mmol, 1.3 equiv.) and anhydrous TEA (0.6 mL, 4.0 mmol, 4.0 equiv.) in 6 mL of THF/DMF (2/1) was added dropwise over a period of 5 min. Reaction was stirred at −78 °C for 5 min. Solvent was then evaporated, and the crude residue purified by normal phase chromatography purification with a CombiFlash Rf Teledyne ISCO apparatus (12 g silica cartridge, solvent A: DCM, solvent B: EtOH, gradient 0 to 20% solvent B in 20 min) to yield the title compound as an off-white solid. (138.6 mg, 60%). ^1^H NMR (400 MHz, DMSO-*d*_6_) (mixture of rotamers) *δ* 8.64 (t, *J* = 7.3 Hz 1H), 8.55 (t, *J* = 7.3 Hz, 1H) 7.86 (m, 1H), 3.91-3.81 (m, 5H), 2.58 (t, *J* = 3.4 Hz, 1H). ^13^C NMR (101 MHz, DMSO-*d*_6_) (mixture of rotamers) *δ* 170.63, 170.28, 169.97, 169.34, 168.42, 168.40, 166.95, 54.98, 54.95, 43.70, 43.53, 25.55, 25.42. *t*_R_: 1.37 min (generic method). ESI-MS for C_9_H_12_N_8_O_2_S: calculated 296.1, found *m/z* 295.1 [M-H]^-^ and 296.1 [M+H]^+^.

**General procedure for the preparation of compounds 2-10.** Under nitrogen 2-((4-chloro-6-methoxy-1,3,5-triazin-2-yl)amino)-N-methylacetamide (intermediate *int_3*) (1 equiv.), the appropriate thiol (1.5 equiv) and TEA (3.0 equiv.) were mixed in a Schlenk flask and then dissolved in dry ACN (4.6 mL). The reaction was heated at 100 °C for 16 h. Solvent was then evaporated, and the crude residue purified by normal-phase chromatography purification with a CombiFlash Rf Teledyne ISCO apparatus to yield the title compound.

**2-((4-methoxy-6-((1-methyl-1H-tetrazol-5-yl)thio)−1,3,5-triazin-2-yl)amino)-N-methylacetamide (2).** The title compound was synthesized following the general procedure with 2-((4-chloro-6-methoxy-1,3,5-triazin-2-yl)amino)-N-methylacetamide (*int_3*) (30.0 mg, 0.13 mmol,1 equiv.), TEA (54.25 μL, 0.39 mmol, 3.0 equiv) and 1-methyl-1H-tetrazole-5-thiol (23.0 mg, 0.2 mmol, 1.5 equiv). The crude was purified by normal phase chromatography purification with a CombiFlash Rf Teledyne ISCO apparatus (12 g silica cartridge, solvent A: DCM, solvent B: EtOH, gradient 0 to 20% solvent B in 20 min) to yield the title compound as an off-white solid. (12.12 mg, 30%). ^1^H NMR (400 MHz, DMSO-*d*_6_)(mixture of rotamers) *δ* 8.38 (q, *J* = 6.0 Hz, 1H), 7.82 (m, 0.5H), 7.67 (s, 0.5H), 4.07 (s, 1.5H), 4.00 (s, 1.4H), 3.79 (d, *J* = 6.2 Hz) 3.78 (s, 1.76H), 3.72 (s, 1.37H), 3.56 (d, *J* = 6.2 Hz, 1H), 2.57 (t, *J* = 4.2 Hz, 3H). *t*_R_: 1.10 min (generic method). ESI-MS for C_9_H_13_N_9_O_2_S: calculated 311.1, found *m/z* 310.1 [M-H]^-^ and 310.1 [M+H]^+^.

**2-((4-methoxy-6-(phenylthio)−1,3,5-triazin-2-yl)amino)-N-methylacetamide (3).** The title compound was synthesized following the general procedure with 2-((4-chloro-6-methoxy-1,3,5-triazin-2-yl)amino)-N-methylacetamide (*int_3*) (30.0 mg, 0.13 mmol,1 equiv.), TEA (54.0 μL, 0.39 mmol, 3.0 equiv) and benzenethiol (22.0 mg, 0.2 mmol, 1.5 equiv). The crude was purified by normal phase chromatography purification with a CombiFlash Rf Teledyne ISCO apparatus (12 g silica cartridge, solvent A: DCM, solvent B: EtOH, gradient 0 to 20% solvent B in 20 min) to yield the title compound as an off-white solid. (13.9 mg, 35%). ^1^H NMR (400 MHz, DMSO-*d*_6_)(mixture of rotamers) *δ* ^1^H NMR (400 MHz, DMSO-*d*_6_) *δ* 8.17 (t, *J* = 6.2 Hz, 0.5H), 8.17 (t, *J* = 6.2 Hz, 0.5H), 7.83 (q, *J* = 4.6 Hz, 1H), 7.59-7.55 (m, 1H), 6.39-6.35 (m, 1.5H), 6.31-6.28 (m, 0.5H), 4.41 (s, 1H), 4.38 (s, 1H), 3.88 (d, *J* = 6.1 Hz, 1H), 3.84-3.81 (m, 4H), 2.58 (m, 3H). ^13^C NMR (101 MHz, DMSO-*d*_6_) (mixture of rotamers) *δ* 180.25, 179.58, 169.25, 169.05, 168.96, 165.76, 165.52, 150.72, 150.68, 142.59, 142.57, 110.76, 110.73, 108.18, 107.98, 54.25, 54.21, 43.74, 43.52, 25.91, 25.82, 25.55, 25.42. *t*_R_: 1.82 min (generic method). ESI-MS for C_13_H_15_N_5_O_2_S: calculated 306.1, found *m/z* 305.1 [M-H]^-^ and 307.1 [M+H]^+^.

**3-((4-methoxy-6-((2-(methylamino)−2-oxoethyl)amino)−1,3,5-triazin-2-yl)thio)benzoic acid (4).** The title compound was synthesized following the general procedure with 2-((4-chloro-6-methoxy-1,3,5-triazin-2-yl)amino)-N-methylacetamide (*int_3*) (30.0 mg, 0.13 mmol,1 equiv.), TEA (54.0 μL, 0.39 mmol, 3.0 equiv) and 3-mercaptobenzoic acid (30.1 mg, 0.2 mmol, 1.5 equiv). The crude was purified by normal phase chromatography purification with a CombiFlash Rf Teledyne ISCO apparatus (12 g silica cartridge, solvent A: DCM, solvent B: EtOH, gradient 0 to 20% solvent B in 20 min) to yield the title compound as an off-white solid. (6.8 mg, 15%). ^1^H NMR (400 MHz, DMSO-*d*_6_)(mixture of rotamers) *δ* 8.15-7.95 (m, 3H), 7.84-7.74 (m, 1H), 7.7 (dt, *J =* 7.7, 1.56 Hz, 1H), 7.6 (dt, *J =* 7.7, 1.56 Hz, 1H), 7.46 (m, 1H), 3.76 (d, *J* = 6.1 Hz, 1.1H), 3.74 (s, 1.2H), 3.72 (d, *J* = 6.2 Hz, 1.9H), 3.69 (s, 1.8H), 2.55 (d, *J* = 4.4 Hz), 2.53 (d, *J* = 4.4 Hz). ^13^C NMR (101 MHz, DMSO-*d*_6_) (mixture of rotamers) *δ* 180.80, 180.46, 169.29, 169.14, 168.90, 168.76, 165.91, 165.65, 137.64, 137.00, 135.96, 135.31, 130.18, 128.73, 128.46, 127.64, 127.42, 54.07, 43.54, 25.53. *t*_R_: 1.2 min (generic method). ESI-MS for C_14_H_15_ N_5_O_4_S: calculated 349.4, found *m/z* 348.4 [M-H]^-^ and 350.4 [M+H]^+^.

**2-((4-((2-bromophenyl)thio)−6-methoxy-1,3,5-triazin-2-yl)amino)-N-methylacetamide (5).** The title compound was synthesized following the general procedure with 2-((4-chloro-6-methoxy-1,3,5-triazin-2-yl)amino)-N-methylacetamide (*int_3*) (30.0 mg, 0.13 mmol,1 equiv.), TEA (54.0 μL, 0.39 mmol, 3.0 equiv) and 2-bromobenzenethiol (37.8 mg, 0.2 mmol, 1.5 equiv). The crude was purified by normal phase chromatography purification with a CombiFlash Rf Teledyne ISCO apparatus (12 g silica cartridge, solvent A: DCM, solvent B: EtOH, gradient 0 to 20% solvent B in 20 min) to yield the title compound as an off-white solid. (18.5 mg, 37%). ^1^H NMR (400 MHz, DMSO-*d*_6_)(mixture of rotamers) *δ* 8.13-8.05 (m, 1H), 7.85-7.72 (m, 2.5H), 7.64-7.57 (m, 0.5H), 7.45 (m, 2H), 3.78 (d, *J* = 5.9 Hz, 1.1H), 3.74 (s, 1.3H), 3.69 (s, 1.7H), 3.67 (d, *J* = 5.9 Hz, 0.9H), 2.57 (t, *J* = 4.5 Hz, 3H). ^13^C NMR (101 MHz, DMSO-*d*_6_) (mixture of rotamers) *δ* 179.95, 179.42, 169.31, 169.18, 168.88, 168.66, 165.90, 165.67, 138.12, 137.87, 133.34, 133.20, 131.79, 131.67, 130.07, 129.79, 129.11, 128.41, 128.22, 54.14, 54.05, 43.52, 43.40, 25.59, 25.54. *t*_R_: 1.86 min (generic method). ESI-MS for C_13_H_14_ BrN_5_O_2_S: calculated 383.0, found *m/z* 382.1 [M-H]^-^ and 384.0 [M+H]^+^.

**2-((4-methoxy-6-(naphthalen-1-ylthio)−1,3,5-triazin-2-yl)amino)-N-methylacetamide (6).** The title compound was synthesized following the general procedure with 2-((4-chloro-6-methoxy-1,3,5-triazin-2-yl)amino)-N-methylacetamide (*int_3*) (30.0 mg, 0.13 mmol,1 equiv.), TEA (54.0 μL, 0.39 mmol, 3.0 equiv), and naphthalene-1-thiol (32.1 mg, 0.2 mmol, 1.5 equiv). The crude was purified by normal phase chromatography purification with a CombiFlash Rf Teledyne ISCO apparatus (12 g silica cartridge, solvent A: DCM, solvent B: EtOH, gradient 0 to 20% solvent B in 20 min) to yield the title compound as an off-white solid. (17.1 mg, 37%). ^1^H NMR (400 MHz, DMSO-*d*_6_)(mixture of rotamers) *δ* 8.22-8.14 (m, 1H), 8.12-8.0 (m,1H),8.04-7.94 (m, 2H), 7.90-7.83 (m, 1H), 7.79-7.74 (m, 0.5H), 7.62-7.49 (m, 3.5H), 3.72 (d, *J* = 4.0 Hz, 1.2H), 3.59-3.52 (m, 3.8H), 2.55-2.5 (m, 3H). ^13^C NMR (101 MHz, DMSO-*d*_6_) (mixture of rotamers) 181.09, 180.59, 169.24, 169.13, 168.90, 168.64, 165.88, 165.63, 135.88, 135.62, 134.35, 134.21, 133.77, 133.66, 131.07, 130.94, 128.66, 128.60, 127.39, 127.21, 126.50, 126.37, 125.91, 125.73, 125.48, 125.44, 125.10, 125.03, 53.93, 53.88, 43.49, 43.30, 40.06, 25.54, 25.52. *t*_R_: 1.92 min (generic method). ESI-MS for C_17_H_17_N_5_O_2_S: calculated 355.4, found *m/z* 354.4 [M-H]^-^ and 356.4 [M+H]^+^.

**2-((4-(3-acetylphenoxy)−6-methoxy-1,3,5-triazin-2-yl)amino)-N-methylacetamide (7).** Under nitrogen 2-((4-chloro-6-methoxy-1,3,5-triazin-2-yl)amino)-N-methylacetamide (*int_3*) (30.0 mg, 0.13 mmol,1.0 equiv.), TEA (54.0 μL, 0.39 mmol, 3.00 equiv), and 1-(3-hydroxyphenyl)ethan-1-one (27.2 mg, 0.2 mmol, 1.5 equiv), were mixed in a schlenk flask and then dissolved in dry ACN (4.6 mL). The reaction was heated at 140 °C for 16 h. Solvent was then evaporated, and the crude residue purified by normal phase chromatography purification with a CombiFlash Rf Teledyne ISCO (12 g silica cartridge, solvent A: DCM, solvent B: EtOH, gradient 0 to 20% solvent B in 20 min) to yield the title compound as an off-white solid. (11.2 mg, 26%). *t*_R_: 1.78 min (generic method). ESI-MS for C_15_H_17_N_5_O_4_S: calculated 331.1, found *m/z* 330.1 [M-H]^-^ and 332.1 [M+H]^+^.

**2-((4-((2,6-dimethylphenyl)thio)−6-methoxy-1,3,5-triazin-2-yl)amino)-N-methylacetamide (8).** The title compound was synthesized following the general procedure with 2-((4-chloro-6-methoxy-1,3,5-triazin-2-yl)amino)-N-methylacetamide (*int_3*) (30.0 mg, 0.13 mmol,1 equiv.), TEA (54.0 μL, 0.39 mmol, 3.0 equiv) and 2,6-dimethylbenzenethiol (27.6 mg, 0.2 mmol, 1.5 equiv). The crude was purified by normal phase chromatography purification with a CombiFlash Rf Teledyne ISCO apparatus (12 g silica cartridge, solvent A: DCM, solvent B: EtOH, gradient 0 to 20% solvent B in 20 min) to yield the title compound as an off-white solid. (19.5 mg, 45%). ^1^H NMR (400 MHz, DMSO-*d*_6_)(mixture of rotamers) *δ* 7.97 (m, 1H), 7.79 (q, *J* = 4.6 Hz, 0.5H), 7.54 (q, *J* = 4.6 Hz, 0.5H), 7.31-7.14 (m, 3H), 3.76 (d, *J* = 5.6 Hz, 1.2H), 3.72 (s, 1.3H), 3.67 (s, 1.7H), 3.61 (d, *J* = 5.4 Hz, 0.8H), 2.56 (t, *J* = 4.1 Hz, 3H), 2.37 (s, 3.4H), 2.33 (s, 2.6H). ^13^C NMR (101 MHz, DMSO-*d*_6_) (mixture of rotamers) *δ* 180.42, 179.86, 169.32, 169.24, 168.96, 168.74, 165.94, 165.71, 143.08, 142.96, 129.81, 129.75, 128.10, 127.92, 127.08, 127.06, 53.98, 53.90, 43.50, 43.31, 25.55, 25.51, 21.59, 21.51, 1.15. *t*_R_: 1.90 min (generic method). ESI-MS for C_15_H_19_N_5_O_2_S: calculated 333.1, found *m/z* 332.1 [M-H]^-^ and 334.1 [M+H]^+^.

**2-((4-((furan-2-ylmethyl)thio)−6-methoxy-1,3,5-triazin-2-yl)amino)-N-methylacetamide (9).** The title compound was synthesized following the general procedure with 2-((4-chloro-6-methoxy-1,3,5-triazin-2-yl)amino)-N-methylacetamide (*int_3*) (30.0 mg, 0.13 mmol,1 equiv.), TEA (54.0 μL, 0.39 mmol, 3.0 equiv) and furan-2-ylmethanethiol (22.8 mg, 0.2 mmol, 1.5 equiv). The crude was purified by normal phase chromatography purification with a CombiFlash Rf Teledyne ISCO apparatus (12 g silica cartridge, solvent A: DCM, solvent B: EtOH, gradient 0 to 20% solvent B in 20 min) to yield the title compound as an off-white solid. (17.3 mg, 43%). ^1^H NMR (400 MHz, DMSO-*d*_6_)(mixture of rotamers) *δ* 8.17 (t, *J* = 6.2 Hz), 8.11 (t, *J* = 6.2 Hz), 7.84 (m, 1H), 7.58-7.55 (m, 1H), 6.39-6.36 (m, 1.4H), 6.31-6.29 (m, 0.5H), 4.40 (s, 1.10H), 4.38 (s, 0.9H), 3.88 (d, *J* = 6.1 Hz, 0.9), 3.84-3.8 (m, 4.1 Hz), 2.59-2.56 (m, 3H). ^13^C NMR (101 MHz, DMSO-*d*_6_) (mixture of rotamers) *δ* 180.25, 179.58, 169.25, 169.05, 168.96, 165.76, 165.52, 150.72, 150.68, 142.59, 142.57, 110.76, 110.73, 108.18, 107.98, 54.25, 54.21, 43.74, 43.52, 25.91, 25.82, 25.55, 25.42. *t*_R_: 1.63 min (generic method). ESI-MS for C_12_H_15_N_5_O_3_S: calculated 309.3, found *m/z* 308.3 [M-H]^-^ and 310.3 [M+H]^+^.

**tert-butyl 4-(4-methoxy-6-((2-(methylamino)−2-oxoethyl)amino)−1,3,5-triazin-2-yl)piperazine-1-carboxylate (10).** Under nitrogen 2-((4-chloro-6-methoxy-1,3,5-triazin-2-yl)amino)-N-methylacetamide *(int_3)* (30.0 mg, 0.13 mmol,1.0 equiv.), TEA (54.0 μL, 0.39 mmol, 3.0 equiv) and tert-butyl piperazine-1-carboxylate (37.3 mg, 0.2 mmol, 1.5 equiv) were mixed in a schlenk flask and then dissolved in dry ACN (4.6 mL). The reaction was heated at 120 °C for 16 h. Solvent was then evaporated and the crude residue purified by normal phase chromatography purification with a CombiFlash Rf Teledyne ISCO (12 g silica cartridge, solvent A: DCM, solvent B: EtOH, gradient 0 to 20% solvent B in 20 min) to yield the title compound as an off-white solid. (4.9 mg, 10%). *t*_R_: 1.84 min (generic method). ESI-MS for C_16_H_27_N_7_O_4_: calculated 381.2, found *m/z* 380.2 [M-H]^-^ and 382.2 [M+H]^+^.

## Experimental biophysical characterization of 1-10 binding to TRAP1 and of TRAP1 ATPase activity

### NMR experiments

All NMR experiments were performed at 298 K using a Bruker 600 MHz FT NMR Avance Neo spectrometer equipped with a QCI CryoProbe ^1^H/19F-13C/^15^N-D-Z with a shielded z-gradient coil and an automated SampleJet sample handling system with temperature control.

A commercially available recombinant human TRAP1 (Enzo Biochem Inc., ADI-SPP-848) was employed for all the experiments. The assay buffer used for all samples consisted of 10 mM HEPES (pH 8.0), 200 mM NaCl, 0.05% Tween 20, 5 mM MgCl₂, 1 mM DTT, and 20% D₂O for the lock signal, with a final DMSO-d₆ concentration of 1%.

**Compounds solubility and aggregation.** Compounds were tested at a concentration of 100 μM in assay buffer in the presence of 200 μM 4-(trifluoromethyl)benzoic acid as an internal reference, using the SPAM filter approach. For each sample, two spectra were acquired.

To evaluate compound identity and solubility, a 1D ^1^H spectrum (Bruker *noesypr* pulse sequence) was recorded. Water suppression was achieved using the standard NOESY presaturation sequence, with the following parameters: 64k data points, spectral width of 30 ppm, 128 scans, acquisition time of 1.835 s, relaxation delay of 4 s, and mixing time of 10 ms.

Compound aggregation was assessed using WaterLOGSY experiments with a 7.5 ms 180° Gaussian-shaped pulse, acquisition time of 0.852 s, mixing time of 1.7 s, relaxation delay of 2 s, and 384 scans.

Prior to Fourier transformation, data were processed using an exponential window function with 0.3 Hz line broadening for both the 1D ^1^H and WaterLOGSY spectra.

**Binding experiments.** Chemical compounds (100 μM) were tested both in the absence and in the presence of 2 μM TRAP1. For each sample, a 1D ^1H *noesypr* experiment was recorded using the same parameters applied for solubility assessment. WaterLOGSY experiments were performed under the same conditions described above, with the exception of an increased number of scans (1024 scans).

**Competition binding experiments.** AMP-PNP (100 μM) was tested in the absence, in the presence of 2 μM TRAP1, and in the presence of 2 μM TRAP1 plus 100 μM test compounds. For all samples, a 1D ^1H *noesypr* spectrum was recorded using the previously described parameters (64 scans), and WaterLOGSY experiments were acquired using 384 scans.

**ATP hydrolysis assay.** Functional assays were performed in an endpoint format in triplicate. ATP (200 μM) was incubated in the absence, in the presence of 0.5 μM TRAP1, and in the presence of 0.5 μM TRAP1 plus 50 or 100 μM compounds in 0.5 mL Eppendorf tubes using a thermomixer set at 37 °C and 350 rpm for 60 min. Reactions were quenched by the addition of 20 μL of 500 mM EDTA (pH 8.0). For each sample, a 1D ^^1^H NMR spectrum was recorded as described above, using 128 scans. To improve separation of ATP and ADP resonances, a Gaussian line broadening of −0.3 Hz with a maximum position (gb) of 0.1 was applied prior to Fourier transformation.

### Microscale thermophoresis

Purified TRAP1 was labeled using the Monolith protein labeling kit RED-NiNTA 2nd generation (Nanotemper) according to the manufacturer’s instructions. The labeled protein was first evaluated in a pre-test assay for optimal fluorescence and to verify the absence of aggregation or capillary adsorption in MST buffer [10 mM HEPES pH8, 200 mM NaCl, 1 mM TCEP, 0.05% Tween-20]. For binding check screening, TRAP1 (25 nM) was incubated with compounds at concentrations of 50 μM in MST buffer for 30 min at room temperature. Protein incubated with DMSO served as the reference control. DMSO concentrations ranging from 1% to 5% were tested. For binding curves measurements, a ten-point 1:2 serial dilution of compound **4** (starting from 2.5 mM) was incubated with 12.5 nM TRAP in MST buffer containing 5% DMSO (final concentration). Samples were acquired in expert mode, and the thermophoretic signal at an MST time of 1.5 s was used for analysis. All measurements were performed using a Monolith NT.115 Pico instrument (NanoTemper). Data analysis was carried out using GraphPad Prism.

### Measurement of TRAP1 ATPase activity

To assess the ATPase activity of TRAP1, a commercially available recombinant human protein (Enzo Biochem Inc., ADI-SPP-848) was used. For each experimental condition, 2 µg of recombinant TRAP1 were added to a reaction buffer containing 50 mM Tris-HCl (Sigma-Aldrich), 150 mM NaCI (Sigma-Aldrich), 10 mM MgCI_2_ (Sigma-Aldrich), pH 7.4. Reactions were prepared at a final volume of 200 μL in light-protected microcentrifuge tubes (Sarstedt) to minimize spontaneous ATP autohydrolysis. Vehicle control (DMSO) (Sigma-Aldrich) or inhibitors were added, and samples were pre-incubated at 37 °C under agitation (200 rpm) for 10 min using a Thermo-Shaker. Following pre-incubation, ATP (AppliChem) was added to a final concentration of 200 µM, and reactions were allowed to proceed at 37 °C, under agitation (200 rpm), for 1 h. The Malachite Green Phosphate Assay Kit (Sigma-Aldrich) was equilibrated to room temperature prior to preparation of the working solution by mixing Reagent A and Reagent B at a 100:1 ratio. After 1 h of incubation, samples were transferred to 96-well plate (Sarstedt), adding 80 μL of sample and 20 μL of the working malachite green solution to each well. Two technical replicates were included for each condition. Plates were gently mixed by tapping and incubated at room temperature for 30 min to allow color development. Absorbance was measured at 620 nm using a Multiskan SkyHigh microplate spectrophotometer (Thermo-Fisher).

A control reaction without TRAP1 was included to correct for ATP autohydrolysis, and its absorbance was subtracted from each sample to specifically determine TRAP1-dependent ATP-hydrolysis. The amount of inorganic phosphate released was calculated using a standard curve prepared with the standards provided in the Malachite Green kit. The nanomoles of phosphate released per sample were normalized first to the reaction time (minutes) and then to the amount of TRAP1 (µg) used in each assay. Results were expressed as micromoles of phosphate released per minute per microgram of TRAP1. Negative control reactions lacking ATP were included to verify the absence of inorganic phosphate contamination.

## Results

### Rational design and computational characterization of an allosteric TRAP1 binder

This work stems from previous achievements, including the identification of an allosteric pocket in the middle-domain of TRAP1 and the development of isoform-selective ligands able to interfere with TRAP1 functions (Figure 1a ligands **5**, **6**, **7,** and **51**).^18^, ^22^

Here, chemical intuition, combined with physical-chemical characterization of the allosteric binding site and regions in the pocket allosterically connected to the ATP-binding site, was employed to merge compounds in Figure 1a into the larger and more drug-like compound **1** (Figure 1a).

Note that all protein structures considered in this work are of TRAP1 in its asymmetric homodimeric closed state, with Mg^2+^ and ATP bound in both protomers’ active sites; for clarity these protein states will be simply termed TRAP1, without mentioning the ions and ligands in the active sites.

The positioning of **1** in the putative allosteric pocket was initially predicted with Glide induced-fit docking calculations (Maestro, Schrödinger Inc. suite) on several initial TRAP1 conformations, obtained from PDB code 4IPE^13^ or from clustering of previous molecular dynamics simulations. These calculations indicated that **1** can adopt different orientations inside the pocket, for example, with the tetrazole pointing towards the protein lumen or towards the surface. Two representative poses, based on ligand conformation and docking score, were selected as the starting point for molecular dynamics (MD) simulations. These poses will be here termed TRAP1(*cryst*)+**1**, depicted in Figure 1b, and TRAP1(*pocket*)+**1** by referring to the correspondent TRAP1 conformations. Details on these protein conformations are available in the Materials and Methods section, whereas the full structures of the protein-ligand complexes are available as Supplementary Files at https://doi.org/10.5281/zenodo.18630105.

**Fig. 2:**
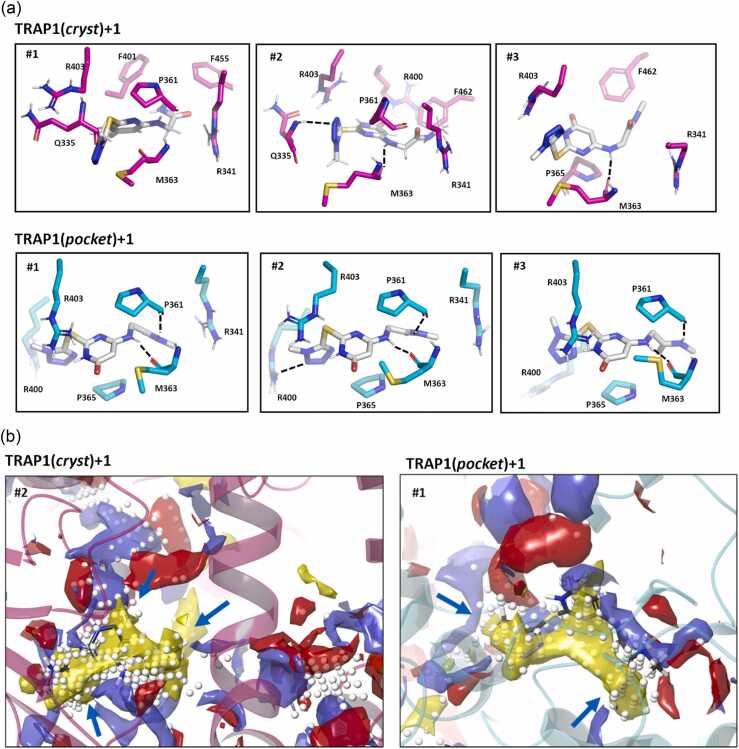
(a) Ligand poses extracted from the three most populated clusters of MD simulations of the two starting poses TRAP1(*cryst*)+**1** (upper panels) and TRAP1(*pocket*)+**1** (lower panels). Compound **1** occupies the pocket described in refs^18^, ^22^ and in the Materials and Methods section. Numbering of protein residues follows that of PDB code 4IPE. Each cluster centroid population is listed in Table S1, and full structures are available as Supplementary Files at https://doi.org/10.5281/zenodo.18630105. (b) Results of pocket analysis conducted with Sitemap (Schrödinger Inc.) on clusters #2 and #1 extracted from TRAP1(*cryst*)+**1** (left) and TRAP1(*pocket*)+**1** (right) meta-trajectories. Pocket analysis on additional cluster centroid structures is in **Figures S2** and **S3**. Areas suitable for H-bond donors, H-bond acceptors, and hydrophobic regions are shown as blue, red, or yellow surfaces, respectively. White spheres represent site points, where volume is available to expand **1**. Light blue arrows highlight areas where the ligand could be extended.

All-atom unbiased classical MD simulations of TRAP1(*cryst*)+**1** and TRAP1(*pocket*)+**1** were run as four independent replicas of 1μs, yielding a 4 μs meta-trajectory for each of the two distinct protein-ligand conformations. Clustering analysis conducted by considering pocket residues and ligand heavy atoms was employed to evaluate the most representative binding poses. Figure 2a depicts ligand conformations obtained from the three most populated clusters in the two meta-trajectories, which cover about 75% of the whole population (Table S1). In the meta-trajectory of TRAP1(*cryst*)+**1** the compound is mainly interacting with Arg341, Gln335, Met363, Pro361 (Figure 2a, upper panels); while in the one resulting from the simulation of TRAP1(*pocket*)+**1** interactions with Pro365, Met363, Pro361, Arg400, and Arg403 are observed (Figure 2a, lower panels). Ligand RMSD analysis along each meta-trajectory (Figure S1) reveals conformational sampling within 0.5-3.0 Å relative to the starting pose, with increased conformational flexibility observed in TRAP1(*cryst*)+**1** trajectory.

Pocket analysis of the structures extracted from MD trajectories clustering suggests considerable scope for chemical modification of **1** through substitutions at the 2, 4 and 6 positions of the central pyrimidone core (Figures 2b and S2-S3). Notably, the tetrazole moiety could be replaced with sterically more demanding hydrophobic cycles, potentially incorporating hydrogen bond donors or acceptors.

### Design and molecular docking of compound 1-derived chemical libraries

**Fig. 3:**
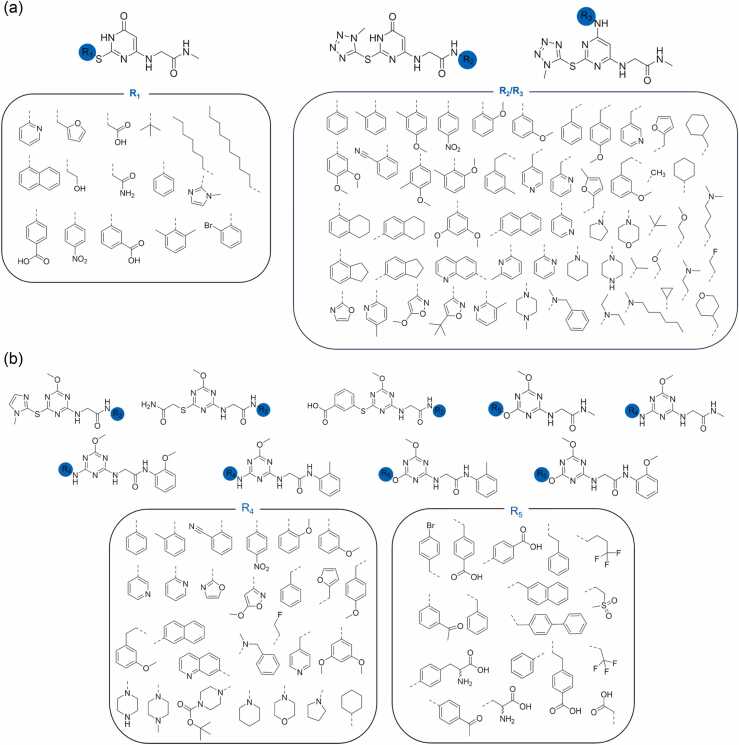
(a) Library of compound **1** derivatives screened with docking calculations; R_1_ substituents are thiols, R_2_/R_3_ are amines. (b) Library of triazine derivatives screened in an additional docking round; R_4_ are amines, R_5_ are alcohols. All original thiol, amine, and alcohol groups are omitted in R derivatives structures except for tertiary/cyclic amines, where the nitrogen is included for clarity. Dashed bonds indicate connection to the rest of the molecule at thiol/alcohol or amine positions.

Prompted by the identification of additional unfilled space around compound **1** in the allosteric pocket and by the possibility of establishing novel protein-ligand interactions, a library of more than 100 synthesizable compounds was built by varying the substituents in 2, 4, and 6 positions of the central pyrimidone core of **1** (Figure 3a).

**Fig. 4:**
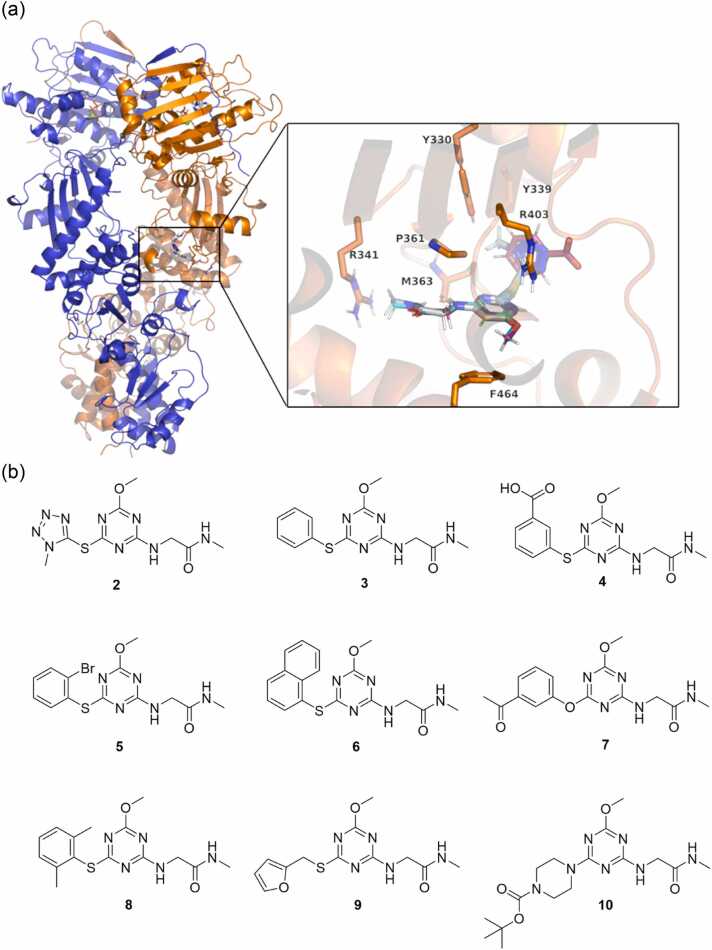
(a) Superimposition of poses obtained from docking calculations of compounds **1**, **2** and **4** in the TRAP1(*cryst*) allosteric pocket. Details on the pocket and on the protein conformation are available in Materials and Methods. (b) Chemical structures of the synthesized triazine compounds **2**-**10**.

Molecular docking calculations of these derivatives on two model TRAP1 model conformations, namely TRAP1(*cryst*), and TRAP1(*MD*) directly obtained from the TRAP1 crystal structure or from previous MD simulations (see Materials and Methods) were performed with Glide (Maestro, Schrödinger Inc. suite). Molecules with the best ranking in both conformations were prioritized for synthesis. A viable synthetic route for **1** was identified, but regioselectivity issues, which lowered the yields emerged, prompting us to slightly modify the core to a 1,3,5-triazinic symmetric core. Also, the hydroxyl group at position 4 was substituted with a methoxy group to avoid the possible coexistence of different tautomeric species in solution. Docking studies confirmed that these modifications did not significantly alter the predicted binding mode and displayed a predicted binding affinity comparable or higher than **1** (Figure 4a, Table S2).

Prompted by these results, additional docking rounds were performed on triazine methoxy analogues of compounds bearing R_1_ and R_2_ substituents shown in Figure 3a, to guide the selection of the most promising candidates. Moreover, new series of compounds featuring amine or alcohol substituents in place of the thiol group, as well as selected di-substituted derivatives, were screened (Figure 3b).

A total of 10 compounds were selected for synthesis based on the visual inspection of the docking poses, their docking scores, and synthetic accessibility. Their structures and best pose docking scores are depicted in Figure 4b and Table S2, respectively. Their preparation is described in the next paragraph.

### Synthesis and chemical characterization of pyrimidone 1 and triazine derivatives 2-10

**Fig. 5:**
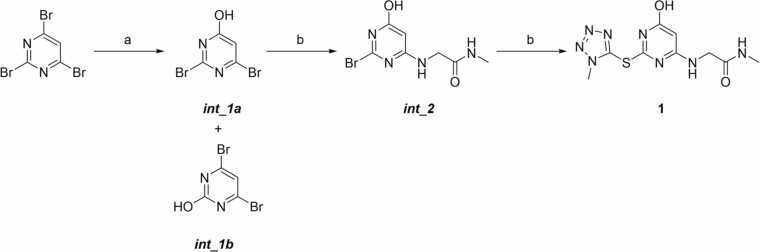
Synthetic routes for the preparation of compounds **1**: (a) NaOH (2M), dioxane, r.t, 4 h, yield: 10%; (b) 2-amino-N-methylacetamide hydrochloride, Et_3_N, CH_3_CN, 90 °C, 16 h, yield: 50%; (c) 1-methyl-1*H*-tetrazole-5-thiol, Et_3_N, 120 °C, 32 h, yields 7%-15%.

The synthetic pathway for the preparation of compound **1** is illustrated in Figure 5. The first step involves the nucleophilic aromatic substitution (S_*N*_Ar) of tribromopyrimidine and hydroxide ions generated from sodium hydroxide in aqueous basic conditions. The reaction yields two regioisomers: the desired product, *int_1a*, where the hydroxyl group is introduced at the 4-position of the pyrimidine ring, and the undesired isomer, *int_1b*, where the hydroxyl group is instead introduced at the 2-position. Both the 4- and 6-positions of the tribromopyrimidine display the highest reactivity in S_*N*_Ar because the negative charge generated during nucleophilic attack is stabilized by the electron-withdrawing nitrogen atoms at the ortho and para positions. However, S_*N*_Ar in position 2 is not negligible, and attempts to optimize the reaction at lower temperatures (e.g., 0 °C) did not succeed, as no product formation was observed. Moreover, their similar polarity and structure made the separation of the two regioisomers particularly challenging via conventional techniques such as flash chromatography or reversed-phase HPLC, leading to a low isolated yield of approximately 10% for *int_1a*. The unambiguous differentiation between the two regioisomers was possible by ¹³C NMR spectroscopy: while the symmetry of *int_1b* results in three distinct carbon signals, the spectra of *int_1a* show that all four carbons resonate at different chemical shifts.

In the second synthesis step, the isolated *int_1a* undergoes a second S_*N*_Ar reaction with 2-amino-N-methylacetamide hydrochloride under basic conditions at 90 °C resulting in the formation of compound *int_2*. In the final step, the remaining bromine atom is displaced via another nucleophilic substitution, this time with the corresponding thiol, at 120 °C yielding the target compound **1**.

**Fig. 6:**
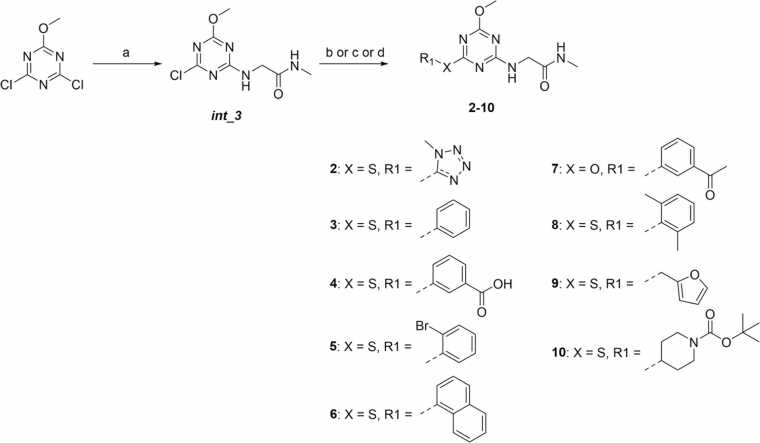
Synthetic routes for the preparation of compounds **2**-**10**. (a) 2-amino-N-methylacetamide hydrochloride, Et_3_N, THF/DMF (2:1), −78 °C, 5 min, yield: 60%; (b) R_1_SH, Et_3_N, 100 °C, 16 h, yields 25- 45%; (c) R_1_OH, Et_3_N, 140 °C, 16 h, yield 26%; (d) R_1_NH_2_, Et_3_N, 120 °C, 16 h, yields 40%.

The triazine derivatives **2-10** were synthesized starting from commercially available 2,4-dichloro-6-methoxy-1,3,5-triazine, which undergoes an initial S_*N*_Ar reaction with 2-amino-N-methylacetamide hydrochloride under basic conditions at a temperature of −78 °C to prevent overreaction and ensure selective substitution (Figure 6). This controlled temperature allows for the formation of intermediate *int_3*, where one chlorine atom is replaced by the amine group.

In the subsequent step**,** *int_3* undergoes a second nucleophilic substitution with the corresponding nucleophile. For thiol-containing nucleophiles, the reaction is carried out at 100 °C to facilitate the substitution. However, for the less nucleophilic alcohol needed for the synthesis of compound **7,** the reaction requires higher temperatures, being conducted at 120 °C and 140 °C, respectively, to achieve the desired product.

All final products and intermediate species were characterized by ^1^H,^13^C NMR and ESI-MS spectrometry.

**Biophysical characterization of 1-10 binding to TRAP1 and evaluation on the ATPase inhibitory activity**

**Fig. 7:**
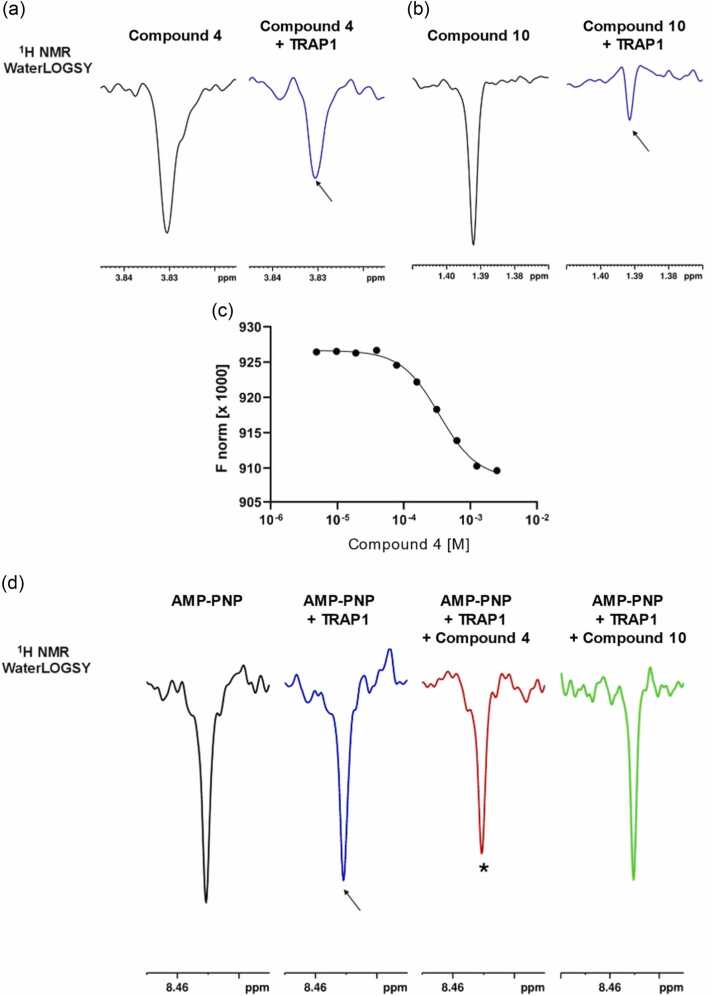
^1^H NMR WaterLOGSY NMR spectra of 100 μM **4**(a) and 100 μM **10**(b) recorded in the absence (black) and presence (blue) of 2 μM TRAP1. Only the WaterLOGSY signals of the methoxy group (compound **4**) and the trimethyl group (compound **10**) are shown. Signal intensity reduction observed upon protein addition (arrows) indicates binding of compounds to TRAP1. (c) Representative titration curve for **4** binding to TRAP1. Labeled TRAP1 (12.5 nM) was incubated with increasing concentrations of **4** for 30 min at room temperature and analyzed by MST. Normalized fluorescence (F_norm×1000) at an MST time of 1.5 s was plotted as a function of compound concentration. Due to the lack of a defined plateau at the highest compound concentrations, the curve was fitted using a four-parameter sigmoidal nonlinear regression (variable slope) to determine an apparent EC₅₀ of 0.43 ± 0.1 mM (mean ± SD, *n* = 3). (d) ^1^H NMR WaterLOGSY spectra of 100 μM AMP-PNP recorded in the absence (black) and in the presence of 2 μM TRAP1 (blue), or 2 μM TRAP1 + 100 μM compound **4** (red) or compound **10** (green). Only the WaterLOGSY signal of the purine proton at position 2 (H2) is shown. The arrow indicates the decrease of the AMP-PNP signal intensity observed upon TRAP1 addition. (*) indicates an additional reduction in the AMP-PNP signal observed upon compound **4** binding.

The initial compound screening was performed by evaluating the ability of the ten compounds to directly bind the purified recombinant protein using two complementary approaches: nuclear magnetic resonance (NMR) and Microscale Thermophoresis (MST). NMR enables the detection of very weak binders (mM affinity) and the assessment of compounds solubility and aggregation under the conditions used for the in vitro experiments^23^ (see Figure S5). MST measures biomolecular interactions by detecting binding-induced changes in thermophoretic movement along a temperature gradient and enables quantification of binding affinities. WaterLOGSY NMR experiments^24^, ^25^ showed that, among the 12 compounds, **4** and **10** clearly bind to TRAP1 (Figure 7a). MST binding check experiments were initially conducted at a concentration of 50 μM, which is below the compounds’ aggregation limit, as assessed by NMR. Under these conditions, only **4** showed reproducible binding to the protein. These data suggest that the interaction between **10** and TRAP1 is weak since only NMR was able to detect it (Figure 7b). Affinity data for **4** binding to TRAP1 were subsequently obtained by MST by titrating a constant concentration of labeled protein (12.5 nM) with increasing concentrations of the ligand (EC_50_ 0.43 ± 0.1 mM, Figure 7c).

To experimentally investigate the proposed allosteric mechanism, NMR displacement assays^26^ in the presence of the non-hydrolysable ATP analogue, AMP-PNP, were performed. These experiments showed that the presence of **4** favors AMP-PNP binding, indicating that the compound does not compete with ATP but modulates its binding through an allosteric mechanism (Figure 7d). In contrast, under the tested conditions, **10** did not affect AMP-PNP binding to TRAP1 in the displacement NMR assay (Figure 7d). Nonetheless, **10** may still act with a distinct allosteric regulatory mechanism.

**Fig. 8:**
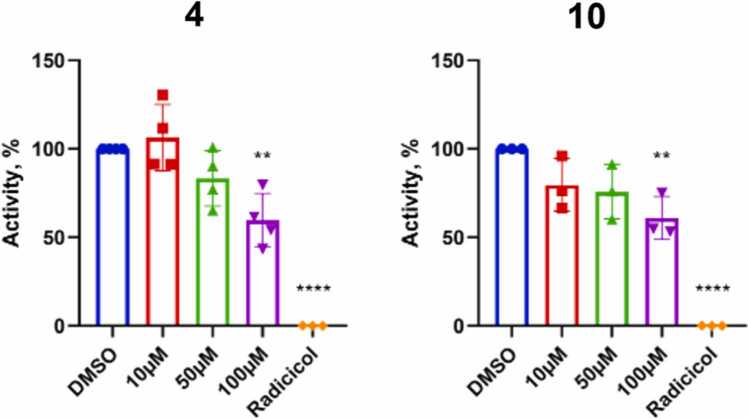
Dose-dependent inhibition of ATP hydrolysis by TRAP1. Inhibitory activity is expressed as a percentage relative to the vehicle control. Radicicol (10 mM), a widely used inhibitor of the HSP90 family of chaperones, was included as a positive control. Data are shown as mean values ±SD (*N* ≥ 3). Statistical significance was assessed by running One-way ANOVA test (**P ≤ 0.01; ***P ≤ 0.0001).

The impact of the synthesized compounds on TRAP1 ATPase activity was also independently evaluated by means of the Malachite Green Phosphate assay. As shown in Figure 8, both **4** and **10** were able to inhibit TRAP1 ATPase activity by 50% at the single dose of 100 μM. In contrast, no measurable changes in ATPase activity were observed following treatment with other inhibitors tested, in agreement with binding analyses.

**Fig. 9:**
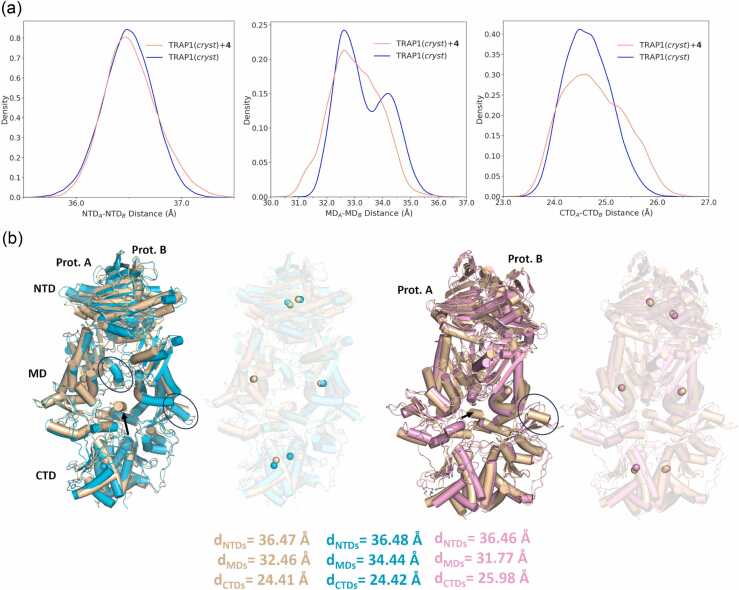
(a) Kernel density estimation plots of inter-protomer domain distances along the TRAP1(*cryst*)+**4** (salmon) or TRAP1(*cryst*) (blue) meta-trajectories. Domains’ centers of mass were taken into account for the computation. NTD comprised residues 101-308; MD 311-571; CTD 587-719. (b) Superimpositions of representative structures extracted from TRAP1(*cryst*) (light brown and cyan) or TRAP1(*cryst*)+**4** (pink) meta-trajectories. The conformation in light brown represents a structure with all inter-protomer distances belonging to the main peaks. The structure in cyan has an MD_A_-MD_B_ distance belonging to the second peak in the TRAP1(*cryst*) distribution; the one in pink shows MD_A_-MD_B_ and CTD_A_-CTD_B_ distance values which are mainly populated by TRAP1(*cryst*)+**4**. Arrows or circles highlight areas in the MD or CTD domain that significantly vary among the conformations considered. Compound **4** is represented in sticks, the calculated domains’ centers of mass are shown as spheres; ATPs and Mg^2+^ ions are omitted. The same superimpositions are also shown with transparent cartoons to highlight the position of the domains’ centers of mass.

Overall, the lack of competition with AMP-PNP, the relatively weak binding affinity observed for **4** (EC₅₀ = 0.43 mM) and the even weaker binding of **10** (detected only by NMR) to TRAP1, despite the approximately 50% inhibition of TRAP1 ATP hydrolysis suggest that these compounds may act as allosteric modulators of TRAP1 activity.

Since the compounds do not directly compete with ATP, their allosteric effects may not directly correlate with their measured binding affinities. It should also be considered that MST and NMR binding experiments were necessarily performed under experimental conditions different from those used in the in vitro functional assay, which may differentially affect stabilization of the orthosteric and/or allosteric pocket, influencing the apparent binding and inhibitory effects. However, despite the differences in the experimental conditions required to carry out the different experiments, it is important to note that all results point into the direction of allosteric binding, influence on the active site, and consequent perturbation of enzymatic activity. Additionally, while the functional in vitro assay supports ATPase inhibition, NMR data confirm this result and add the interesting observation that nucleotide binding affinity may actually be influenced by the presence of the allosteric ligand. Overall, despite the differences in outcomes that need to be critically considered, the use of complementary techniques permits us to develop an exhaustive model for the activities of designed compounds.

To further support the inhibitory activity of the best identified candidate inhibitor **4** in the NMR binding conditions, TRAP1 ATP hydrolysis in the absence and presence of this compound was monitored by NMR, showing an approximately 25% inhibition at a concentration of 100 μM (Figure S5).

### Computational model of the mode of action of compound 4

At this point, we turned back to computational techniques to characterize the effects of the allosteric ligand on the global dynamics of the protein. All-atom unbiased classical MD simulations of TRAP1(*cryst*) in the presence and absence of **4** were carried out as four independent replicas of 500 ns, yielding a total of 2 μs meta-trajectory for each of the systems. Interestingly, analyses of inter-protomer domain distances and contacts along the trajectories highlighted a significant variation in the TRAP1 conformational ensemble following **4** binding to the allosteric pocket. This is apparent especially in alterations of the MDs and CTDs dynamics. While just a slight distribution shift towards higher distances/less contacts is observed for NTDs, the 2 peaks distribution of TRAP1(*cryst*) MD_A_-MD_B_ distances turns into a broader, one-peak distribution shifted to lower distances for TRAP1(cryst)+**4,** but the distribution of interprotomer contacts shifts to lower values (Figure 9 and S4). In the presence of **4**, the distribution of CTD_A_-CTD_B_ distances broadens while shifting to higher distances but is accompanied by an increase in the interprotomer contacts. Similarly, the differential activating or inhibiting effects of Hsp90 point mutations have been previously correlated to an increase or decrease in the number of inter-protomer contacts.^27^

## Discussion

Protein homeostasis is essential for cellular fitness, and the Hsp90 family sits at the center of this network by using ATP-driven conformational cycling to fold and tune the activity of a broad repertoire of client proteins, many of which are dysregulated during neoplastic progression when Hsp90 chaperones are markedly induced. This centrality has motivated intense efforts to inhibit Hsp90 for therapeutic purposes, yet despite extensive clinical testing of active-site competitors, efficacy has been limited by toxicity, lack of isoform discrimination, and induction of the pro-survival heat shock response. These limitations underscore the need for isoform-selective chemical tools that can provide a path toward better-tolerated therapeutic strategies. Importantly, such compounds could also enable the mechanistic dissection of compartment-specific chaperone networks and the comprehension of their regulation under pathologic conditions. TRAP1, the mitochondrial Hsp90, is particularly attractive in this context because its clients and biological roles are restricted to mitochondria, and therefore they are distinct from those of cytosolic Hsp90 and ER-resident GRP94. Mechanistically, TRAP1 further differs in that its closed, active dimer is intrinsically asymmetric at the client-binding middle–C-terminal domain interface, enforcing sequential ATP hydrolysis and an asymmetry flip that can reorganize client-binding determinants. Our findings build on this conceptual framework by showing that rationally designed, drug-like allosteric ligands targeting a TRAP1-specific middle-domain pocket can indeed impact the chaperone’s mechanisms. In particular, the lead compound binds TRAP1 with measurable affinity, alters nucleotide engagement without displacing ATP, and modulates ATP turnover and hydrolysis. It is worth noting at this point that our designed inhibitors achieve a limited inhibition of ATP hydrolysis, an aspect that can be determined by several factors. First, the generated inhibitors were developed to directly influence ATPase by binding at a distal site, but no optimization steps in the direction of increasing functional impact were carried out at this stage. Second, we could speculate that the residence times of the ligands on the protein may be limited: the allosteric site is in fact substantially exposed and large, thus very different from the classical well-defined active site grooves that are typically occupied by orthosteric inhibitors, making target engagement more transient and limiting the effects of the ligands. Third, an intrinsic difficulty in optimizing allosteric ligands resides in the fact that experimental assays report measurement of a biochemical parameter, ATPase in this case, that involves the distal active/orthosteric site. While on the one hand this may not directly correlate with binding at the allosteric site as discussed above, it should be noticed that binding is only one aspect of the interplay of structural and conformational dynamic perturbations determined by the cross-talk between the allosteric ligand and the protein. All these factors, combined to the intrinsic limitations of MD-based models of allostery and functional activity, make the derivation of structure-activity relationships for allosteric ligands more complex than for orthosteric ones.

However, it is tempting to speculate here that in a highly regulated system such as the chaperone machinery (in the cytosol, ER, or the mitochondria), even small perturbations in the dynamics of a central hub may significantly impact the functional outcome, as observed previously.^28^, ^29^

Our molecular dynamics analyses reveal that the effect of ligand biding is to reshape the TRAP1 conformational landscape. Specifically, allosteric binding drives a shift of the protein’s dynamic states away from the native closed state toward an alternative ensemble characterized by destabilization of interdomain contacts within the middle and C-terminal regions. We hypothesize that such distortion of the native ensemble is likely to determine functional consequences. The region where the conformational perturbation takes place overlaps with the established client-binding interface, suggesting a direct mechanistic link. In this framework, the ligand may interfere with client engagement by competing for the same surface as that required for substrate recognition. Beyond direct competition, the ligand-induced rewiring of TRAP1 dynamics may further bias the chaperone toward conformational states that are not optimal for client recruitment. Together, these findings point to a model in which ligand binding perturbs the conformational ensemble of TRAP1, thereby modulating its capacity for client binding and remodeling at the level of both structure and dynamics.

At this point, it is also tempting to speculate that the allosteric ligand may impact on the intrinsic asymmetry that underlies ATP processing by TRAP1. In the deterministic model of TRAP1 ATPase activity, hydrolysis is initiated in a single protomer, followed by a conformational rearrangement of the middle and C-terminal domains that primes the second protomer for the subsequent reaction.^14^ The localization of the allosteric perturbator at the region where this conformational switch is triggered raises the possibility that ligand binding directly reshapes the mechanisms of this ATPase cycle.

Mechanistically, such an allosteric effector could act at multiple levels. Kinetically, ligand engagement may modify the energy landscape, altering the height of the barriers that separate functional conformational states and thereby modulating the rate or directionality of interprotomer communication. Thermodynamically, it may bias the equilibrium distribution of ensembles, stabilizing distinct conformers that differ from the native protein in terms of catalytic competence or partner interaction.

In this framework, the ligand plays an intricate number of roles, not only by engaging TRAP1 and directly competing with client binding but rewiring the dynamic asymmetry of the TRAP1 cycle, redefining both its catalytic progression and presentation to partners. Ultimately, this would translate into the remodeling of TRAP1 interaction networks.

Together, these results establish that transient asymmetric conformations of TRAP1 are actionable for the design of new ligands and suggest that targeting allosteric control points rather than the conserved ATP pocket may offer a general strategy to achieve selective modulation of Hsp90-family chaperones.

## CRediT authorship contribution statement

**Elisa Romeo:** Validation, Methodology, Investigation. **Giorgio Colombo:** Writing – review & editing, Writing – original draft, Supervision, Methodology, Investigation, Data curation, Conceptualization. **Martina La Spina:** Supervision, Methodology, Investigation, Formal analysis, Data curation. **Francesca Sbuelz:** Methodology, Investigation. **Claudio Laquatra:** Methodology, Investigation, Data curation. **Marina Veronesi:** Writing – original draft, Methodology, Investigation, Data curation. **Marco Lolicato:** Writing – original draft, Methodology, Investigation. **Federica Guarra:** Writing – review & editing, Writing – original draft, Visualization, Supervision, Methodology, Investigation, Data curation, Conceptualization. **Cristina Arrigoni:** Writing – original draft, Methodology, Investigation, Data curation. **Denis Komarov:** Methodology, Investigation. **Andrea Ciamarone:** Writing – original draft, Methodology, Investigation. **Elisabetta Moroni:** Writing – original draft, Validation, Methodology, Investigation, Conceptualization. **Stefano A. Serapian:** Methodology, Investigation, Data curation. **Luca Torielli:** Visualization, Methodology, Investigation, Data curation. **Stefania Girotto:** Writing – original draft, Methodology, Investigation, Conceptualization. **Viola Previtali:** Writing – original draft, Supervision, Methodology, Investigation, Conceptualization. **Natasha Margaroli:** Methodology, Investigation. **Andrea Rasola:** Writing – review & editing, Writing – original draft, Methodology, Conceptualization.

## Declaration of Competing Interest

The authors declare the following financial interests/personal relationships, which may be considered as potential competing interests: Giorgio Colombo reports financial support and administrative support were provided by the AIRC Foundation for Cancer Research. If there are other authors, they declare that they have no known competing financial interests or personal relationships that could have appeared to influence the work reported in this paper.

## Data Availability

The data are shared on zenodo. links to dois are available in the paper.
