# Supplementary material for: New allosteric modulators of molecular chaperone TRAP1 from the integration of computational biology, medicinal chemistry, and biophysics
Source: Cell Stress Chaperones. 2026 Feb 26;31(2):100162. doi: 10.1016/j.cstres.2026.100162 (PMC12995838; doi:10.1016/j.cstres.2026.100162)
Supplement: Supplementary file 1 — Supplementary material [file mmc1.pdf]

# Supporting Information − New Allosteric Modulators of Molecular Chaperone TRAP1 from the Integration of Computational Biology, Medicinal Chemistry, and Biophysics

Federica Guarra,[1,*] Denis Komarov,[2] Andrea Ciamarone,[3] Luca Torielli,[1] Viola Previtali,[3] Natasha Margaroli,[4] Elisa Romeo,[4] Martina La Spina,[2] Francesca Sbuelz,[2] Claudio Laquatra,[2] Marina Veronesi,[4] Marco Lolicato,[5] Cristina Arrigoni,[5] Elisabetta Moroni,[6] Stefano A. Serapian,[1] Stefania Girotto,[4] Andrea Rasola,[2] Giorgio Colombo[1,*]

1) University of Pavia, Department of Chemistry, via Taramelli 12, 27100 Pavia (Italy).
2) Department of Biomedical Sciences, University of Padova, Padova, Italy
3) D3 PharmaChemistry, Istituto Italiano di Tecnologia, via Morego 30, 16163, Genoa, Italy
4) Structural Biophysics Facility, Istituto Italiano di Tecnologia, via Morego 30, 16163, Genova, Italy
5) Department of Molecular Medicine, University of Pavia, Via Ferrata 2, 27100 Pavia, Italy
6) National Research Council of Italy, Istituto di Scienze e Tecnologie Chimiche "Giulio Natta" (SCITEC-CNR), Via Mario Bianco 9, 20131 Milano, Italy

*corresponding authors: federica.guarra@unipv.it; g.colombo@unipv.it

**Table S1.** Fraction of frames (Population) belonging to each cluster extracted from MD simulations of TRAP1-compound **1** complexes starting from two distinct protein-ligand conformations, namely TRAP1(*cryst*)+**1** and TRAP1(*pocket*)+**1** as described in the main text. Details on the clustering algorithm employed are in the Materials and Methods section. Structures of all six cluster centroids are available as Supplementary Files at https://doi.org/10.5281/zenodo.18630105.

| **TRAP1(*cryst*)+1** | | **TRAP1(*pocket*)+1** | |
|---|---|---|---|
| **Cluster** | **Population** | **Cluster** | **Population** |
| #1 | 0.278 | #1 | 0.335 |
| #2 | 0.238 | #2 | 0.253 |
| #3 | 0.233 | #3 | 0.163 |
| #4 | 0.142 | #4 | 0.148 |
| #5 | 0.104 | #5 | 0.056 |
| #6 | 0.005 | #6 | 0.045 |

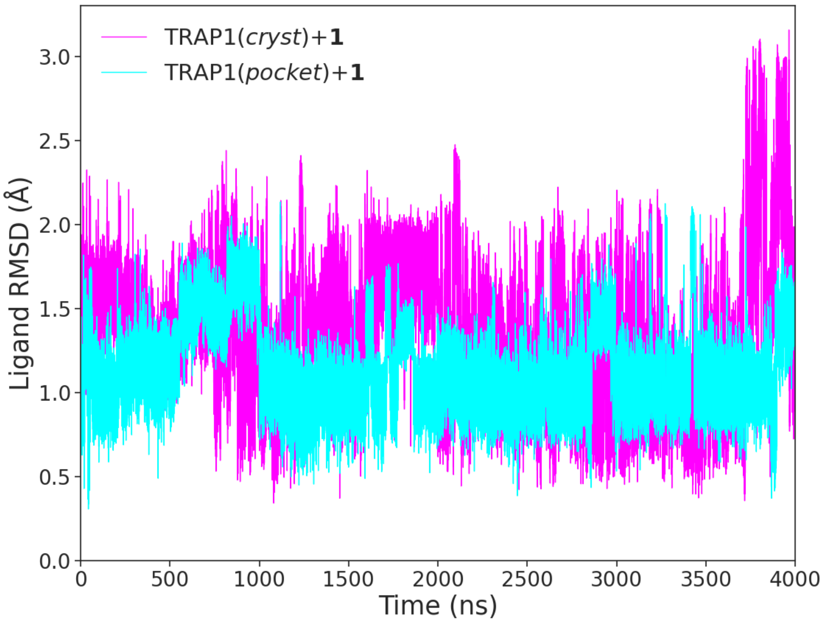


**Figure S1.** Compound **1** Root Mean Square Deviation (RMSD) along the whole MD meta-trajectories of TRAP1(*cryst*)+**1** (magenta) and TRAP1(*pocket*)+**1** (cyan). The reference structure is the ligand starting structure for each MD trajectory as obtained from Induced-Fit Docking calculations; only heavy atoms of compound **1** are taken into account for the RMSD calculation.

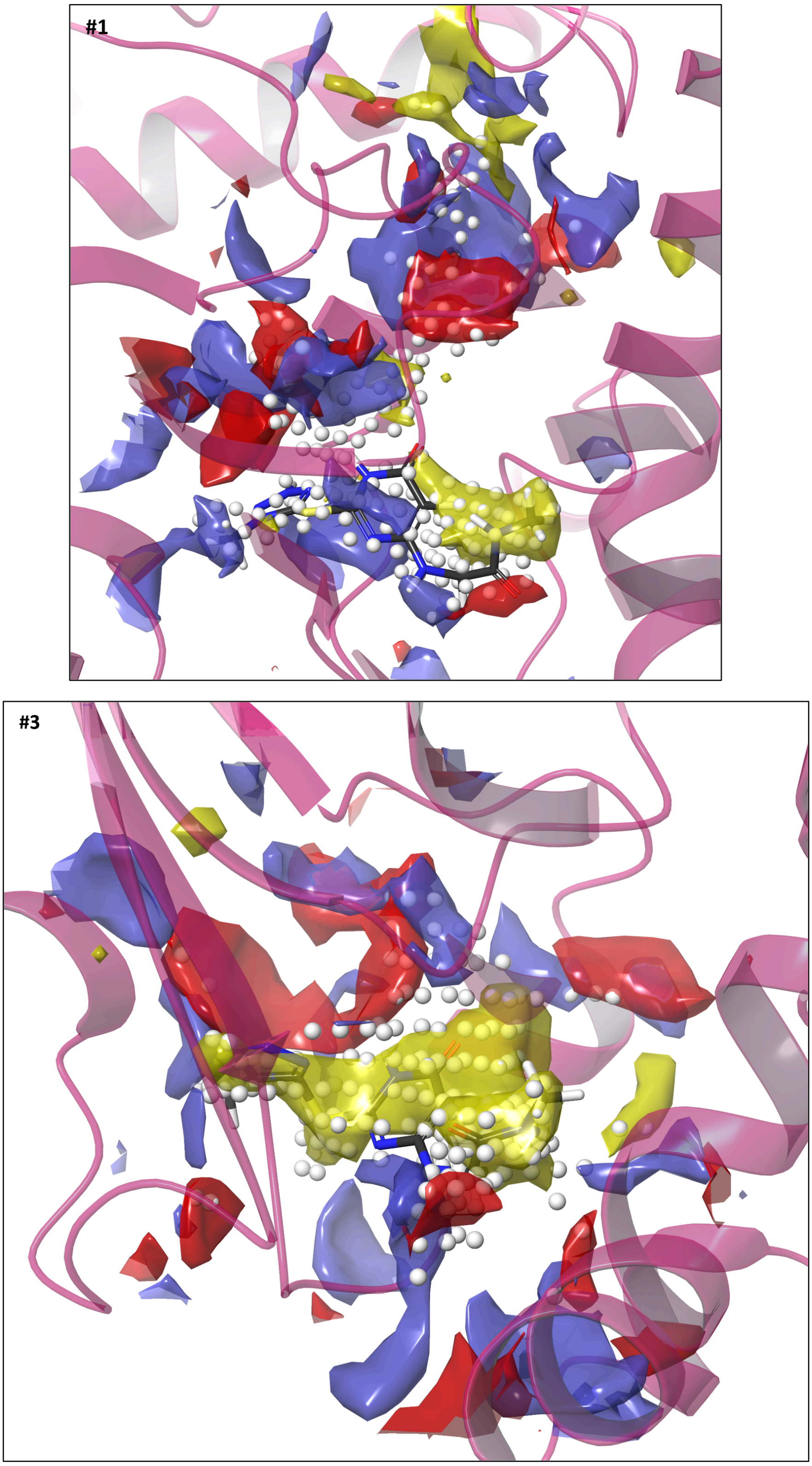


**Figure S2.** Results of Sitemap pocket analysis on cluster centroids #1 and #3 extracted from clustering analysis of MD simulations starting from TRAP1(*cryst*)+**1** pose. Areas suitable for H-bond donors, H-bond acceptors and hydrophobic regions are shown as blue, red or yellow surfaces respectively. White spheres represent volume available within the pocket. Details on the clustering method are in the main text.

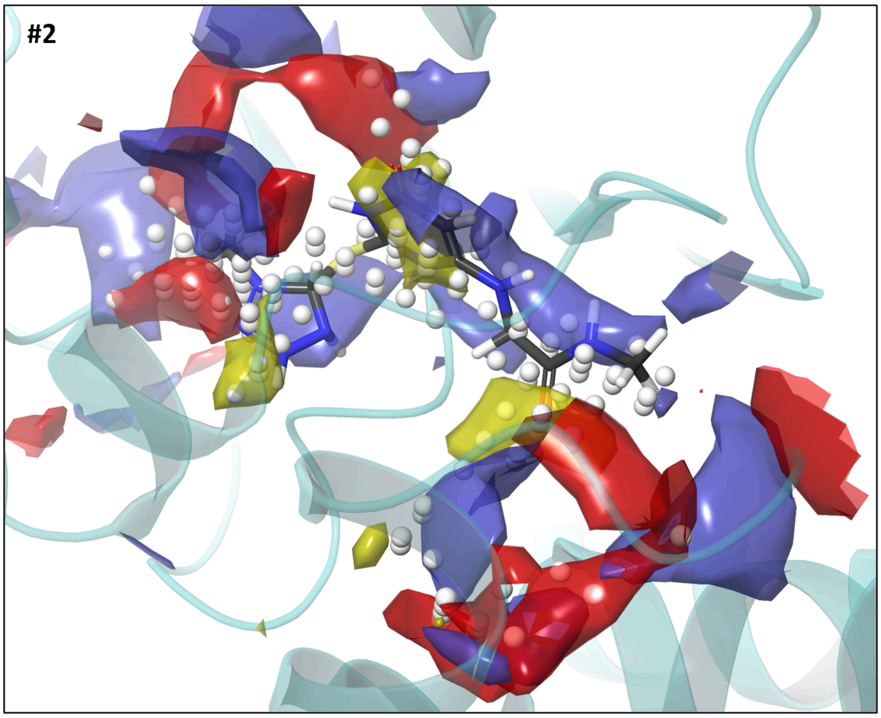


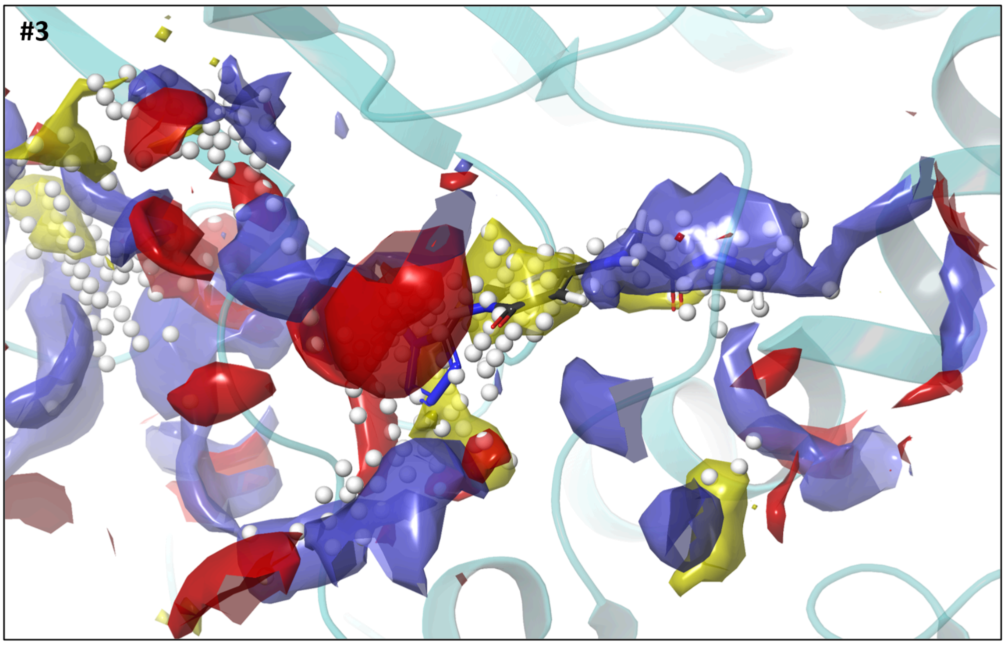


**Figure S3**. Results of Sitemap pocket analysis on cluster centroids #1 and #3 extracted from clustering analysis of MD simulations starting from TRAP1(pocket)+**1** pose. Areas suitable for H-bond donors, H-bond acceptors and hydrophobic regions are shown as blue, red or yellow surfaces respectively. White spheres represent volume available within the pocket. Details on the clustering method are in the main text.

**Table S2**. Docking score of compounds **1-10** in the previously identified allosteric site of two TRAP1 conformations. Results refer to the highest ranking pose obtained from Glide docking calculations in XP mode. Details on the allosteric pocket and TRAP1 conformations are in the Materials and Methods section.

| **Compound** | **Docking score (kcal mol$^{-1}$)** | |
|---|---|---|
| | **TRAP1(*cryst*)** | **TRAP1(*MD*)** |
| **1** | -7.47 | -4.83 |
| **2** | -7.48 | -5.47 |
| **3** | -7.38 | -5.85 |
| **4** | -7.00 | -6.69 |
| **5** | -7.26 | -5.39 |
| **6** | -7.08 | -6.35 |
| **7** | -8.33 | -7.54 |
| **8** | -7.27 | -6.52 |
| **9** | -7.36 | -6.11 |
| **10** | -7.62 | -5.09 |

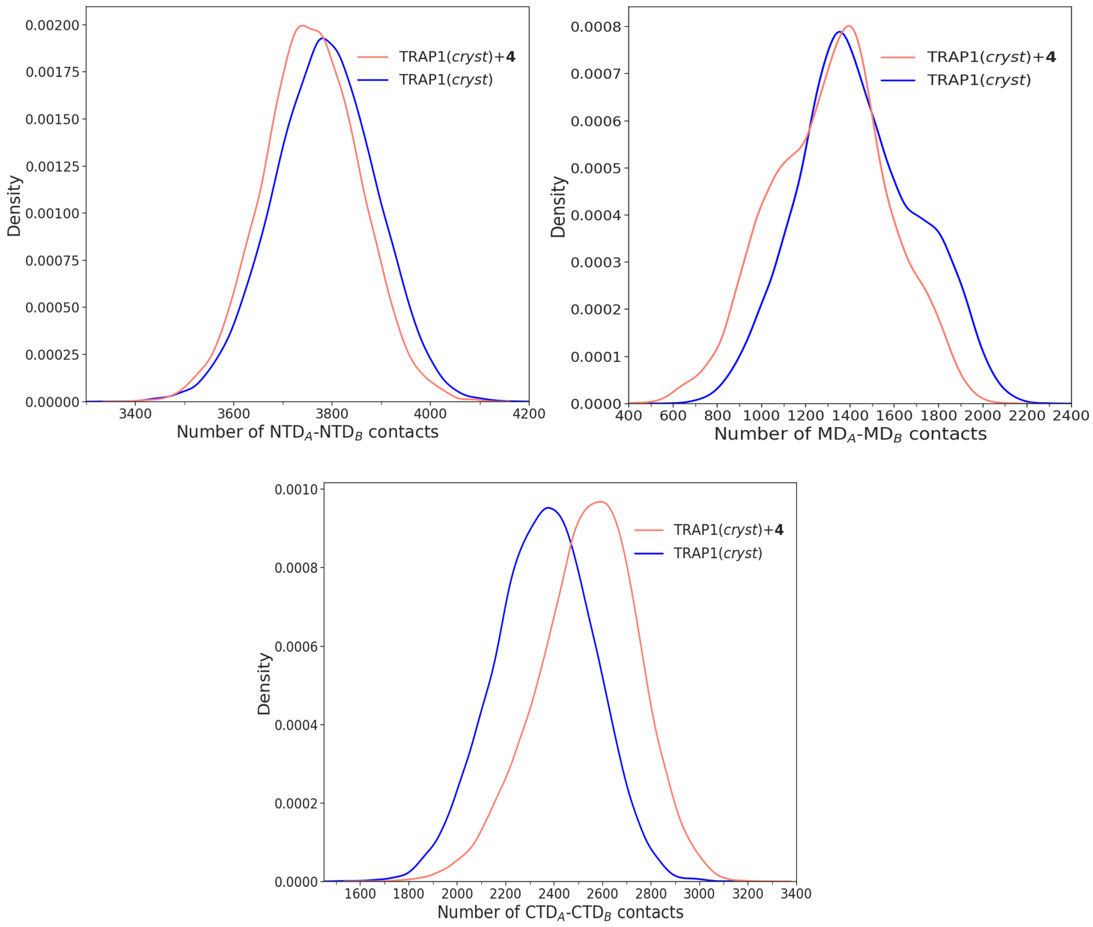


**Figure S4.** Kernel Density Estimation Plots of protomer A- protomer B contacts along the TRAP1(*cryst*)+**4** (salmon) or TRAP1(*cryst*) (blue) meta-trajectories. Only heavy atoms were considered, and the distance cutoff was 7 Å. NTD comprised residues 101-308; MD 311-571; CTD 587-719.

## Compound 4

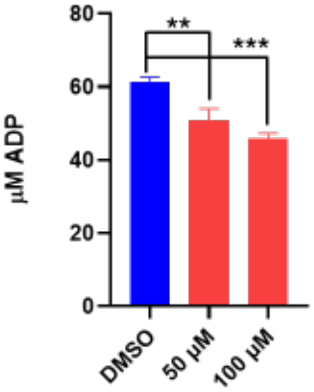


| Compound | [Cpd] μM | % inhibition |
|---|---|---|
| 4 | 50 | 17.4 ± 5.2 |
| | 100 | 25.4 ± 2.3 |

**Figure S5**. The plot shows the amount of ADP measured by $^{1}$H NMR in the presence of DMSO (blue, control) and in the presence of 50 and 100 μM compound **4**. ADP levels decrease proportionally with increasing compound concentration, indicating inhibitory activity of compound **4**. Data are reported as mean ± SD (N ≥ 3). Statistical significance was assessed using a one-way ANOVA (**p ≤ 0.01; ***p ≤ 0.0001). The table on the right reports the inhibitory activity of compound **4** at the two tested concentrations, expressed as a percentage relative to the vehicle control.
